# A Survey of Computer Vision Methods for 2D Object Detection from Unmanned Aerial Vehicles

**DOI:** 10.3390/jimaging6080078

**Published:** 2020-08-04

**Authors:** Dario Cazzato, Claudio Cimarelli, Jose Luis Sanchez-Lopez, Holger Voos, Marco Leo

**Affiliations:** 1 Interdisciplinary Center for Security, Reliability and Trust (SnT), University of Luxembourg, 1855 Luxembourg, Luxembourg; claudio.cimarelli@uni.lu (C.C.); joseluis.sanchezlopez@uni.lu (J.L.S.-L.); holger.voos@uni.lu (H.V.); 2 Institute of Applied Sciences and Intelligent Systems, National Research Council of Italy, 73100 Lecce, Italy; marco.leo@cnr.it

**Keywords:** computer vision, 2d object detection, unmanned aerial vehicles, deep learning

## Abstract

The spread of Unmanned Aerial Vehicles (UAVs) in the last decade revolutionized many applications fields. Most investigated research topics focus on increasing autonomy during operational campaigns, environmental monitoring, surveillance, maps, and labeling. To achieve such complex goals, a high-level module is exploited to build semantic knowledge leveraging the outputs of the low-level module that takes data acquired from multiple sensors and extracts information concerning what is sensed. All in all, the detection of the objects is undoubtedly the most important low-level task, and the most employed sensors to accomplish it are by far RGB cameras due to costs, dimensions, and the wide literature on RGB-based object detection. This survey presents recent advancements in 2D object detection for the case of UAVs, focusing on the differences, strategies, and trade-offs between the generic problem of object detection, and the adaptation of such solutions for operations of the UAV. Moreover, a new taxonomy that considers different heights intervals and driven by the methodological approaches introduced by the works in the state of the art instead of hardware, physical and/or technological constraints is proposed.

## 1. Introduction

Unmanned Aerial Vehicles (UAVs), also called Unmanned Aircraft Systems (UASs), and commonly known as *drones*, are aircraft that fly without a pilot on-board. This places numerous advantages in terms of pilot safety, training, and aircraft costs and sizes, with a huge impact in the range of possible applications. Numbers behind the UAV industry are impressive: Value Market Research estimated that the market for VTOL (Vertical Take-Off and Landing) UAVs will touch around USD 10,163 M by 2024 [1]. Another report from PwC [2] estimates that, in 2030, there will be 76,000 drones operating in the UK skies, involving a total of 628,000 jobs. These forecasts will imply, still in the UK, an increase in GDP of 42 bn*£* and net savings for the UK economy of 16 bn*£*. Finally, an EU report of 2016 [3] estimated an economic impact exceeding €10 bn per year within 20 years. As a consequence, both research and industry are investigating the challenges involved in the manufacturing as well as the design of hardware, software, sensors and algorithms to guarantee the UAV operability and to extend its range to unseen scenarios.

In fact, UAVs already achieved an unprecedented seen level of growth in many civil and military application domains [4]. UAVs can be remotely controlled by a pilot or can fly autonomously. In the former scenario, the pilot is on land- or sea-based ground control station (GCS) for human control. The simplest GCS consists of a remote controller with an optional screen, even in the form of a tablet. In the latter scenario, instead, a pre-scheduled flight plan and a dynamic automation system are necessary. The *Holy Grail* for the involved actors in this revolution is the achievement of fully autonomous operational capabilities to flight over and understand real and complex scenarios. In such an ideal system, a high-level and possibly on-board module is exploited to build semantic knowledge used to reach the application goal. The semantic knowledge leverages low-level software components that extract information concerning what is sensed.

Both exteroceptive and proprioceptive sensors are used to obtain situational and self-awareness [5]. From the beginning, UAVs were equipped with sensors such as Global Positioning System (GPS) and Inertial Navigation System (INS) to provide position and orientation in space, but they come with serious drawbacks. The precision of the GPS depends on the general number of available satellites; moreover, urban canyons and indoor navigation can seriously compromise the navigation. INS, instead, suffers from integration drift with acceleration and angular velocity error accumulation, requiring a correction scheme.

Presently, the software and hardware advancements in embedded systems and the corresponding miniaturization have led to performing low-cost sensors and Inertial Measurement Units (IMUs) that can extract useful information on-board, such as force, angular rate, and orientation. Many approaches and configurations have been also proposed to get significant knowledge of the environment from data acquired by consumer RGB cameras, depth sensors, LiDAR (Light Detection and Ranging), and event-based cameras. Complex and complete sensor fusion suites that merge multiple data have been introduced too.

For each sensor that can be potentially mounted on-board of the UAV, a plethora of works and applications have been proposed. Independently from the employed sensor, where evidently each one comes with own pro and cons, and/or each sensor is better performing in a specific scenario, all of the approaches share the goal of providing meaningful input for the high-level components. Undoubtedly, computer vision can provide a critical contribution to the autonomy of the UAVs and their operational capabilities [6].

Typical UAV operations are surveillance, person tracking, path planning, obstacle avoidance (see Section 2): all of these tasks strongly relies on the detection of one or more domain-related objects. Object detection has then been widely investigated since the beginning of computer vision. Historically, detecting objects in images taken from a camera has represented one of the first computer vision tasks ever: early works are dated to 1960s [7], and a kick-off work that is famous (and considered quite optimistic) in the computer vision community *Summer Vision Project* is dated 1966 [8]. Henceforth, the possibility of detecting and recognizing classes of objects has been considered to be the first component of any artificial intelligence system, and many proposed theoretical techniques in the computer vision community have been applied to such a task.

If many impressive results have been achieved even outperforming human-level performance in object detection, there is still a gap to be filled when the problem is translated to the specific case of aerial robotics. Different challenges are involved in terms of performance, scene, object classes, point of view, perspective, data acquisition, and so on. Moreover, if many datasets are available for object recognition task, they cannot be directly employed in the case of UAVs since the scenes are different from the operational working conditions of the UAV (e.g., indoor, cluttered environments, cameras placed at ground level). Finally, computational constraints and/or communication schemes are an important issue to be addressed.

Very precise surveys on the role of computer vision for the autonomous operation of UAV exist. They focus on high-level operations [6,9], they consider only a specific altitude and/or imaging type [10,11], a technology [12], or a precise use-case [5,13,14,15,16]. It can be observed how, in these very important surveys, the computer vision point of view has been only partially considered. This has been the first motivation behind this work. This work investigates the object detection problem for the specific case of the UAVs from RGB and 2D object detection, introducing also main concepts and references for mixed sensor suites and/or 3D object detection. The second motivation is in the need for a different categorization of the UAVs: there are many valid classification schemes based on characteristics as the mass, size, mission range, operation heights, level of autonomy, flying principle, operation condition [17]. Anyway, concerning the height, it is common to classify UAVs in intervals of 150–300 m, 3000, 5000 and even 20,000 m, always starting from 50 m. From a computer vision point of view, we think that this scheme is not sufficient to understand the methodologies that are applied to address the object detection problem depending on the operational height.

Thus, a conceptual approach that takes into account the operations carried out at very different heights has not been proposed yet. To the best of our knowledge, we present the first work that classifies object recognition methods for the case of UAVs that considers different heights intervals, whose definition is given by the methodological approaches introduced by the works in the state of the art instead of hardware, physical and/or technological constraints. At the same time, our proposal is well-integrated with active EU rules and procedures for the operation of unmanned aircraft. Moreover, how specific state of the art deep learning architectures are adapted for the case under consideration is discussed, and the main applications that introduce their own scientific and technological peculiarities are illustrated. Finally, the different datasets specifically designed to evaluate object detection from aerial views are reported and detailed.

Summing up, the main contributions of this work are:

- an update of dominant literature aiming at performing object detection from UAV and aerial views;
- a taxonomy for the UAV based on the computer vision point of view, that considers how the same problem can drastically change when it is observed from a different perspective;
- a critical discussion of the actual state of the art, with particular attention to the impact of deep learning.

The manuscript is organized as follows: first of all, the role of object detection from UAVs in terms of higher-level operations is illustrated in Section 2, also introducing some important sensors employed in the state of the art besides RGB cameras. In Section 3, an introduction of the terminology behind the object detection problem is given, together with the taxonomy used to classify the works object of the review. In Section 4, the main backbone networks for both classic and mobile cases are described. Works of each introduced category are detailed in Section 5, Section 6 and Section 7, analyzing eye level view, low and medium heights, and aerial imaging, respectively. An overall discussion is given in Section 8, while Section 9 has the conclusion.

## 2. Background on UAVs

The terminology to refer to UAVs is wide and sometimes confusing. UAVs started their life in the military sector, distinguishing between two technical terms: (1) Unmanned Aerial Vehicle (UAV), that designates the aircraft vehicle including all its on-board payload; and (2) Unmanned Aerial System (UAS), that gathers all related elements, including the UAV itself, the ground segment (i.e., the Ground Control Station, GCS), and the communication segment. These terms are not only still in use in the military sector [18], but they are also currently widely used and accepted in the civilian sector. Early civilian applications coined the terms Remotely Piloted Aircraft (RPA) and Remotely Piloted Aerial System (RPAS), to create a gap with military UAVs/UASs, and to emphasize the existence of a remote pilot that assumes the legal responsibility of the operation of the UAV. Nevertheless, these terms are currently obsolete. Researchers in robotics usually employ the term Aerial Robot [19], since it emphasizes the fact that it carries out an autonomous operation, being the role of the human remote pilot just a mere supervisor to take over control in case of unexpected and unrecoverable failure.

Besides, the word drone has been widely used by non-experts and media, without technical distinctions between the aerial and ground segment. Finally, in the late civilian regulations [20], the terms Unmanned Aircraft and Unmanned Aerial System (UAS) are employed, being inclusive with both autonomous and remotely piloted UAVs. In this paper, we use the term UAV in the broader possible sense, including any kind of aerial aircraft and its on-board payload. The ground and communication segments are omitted for simplicity, being aware that in some of the presented works, on ground computations are carried out.

There exist different kinds of UAVs, that can be classified according to several criteria [21,22] such as lifting system, size, maximum take-off weight (MTOW), payload weight, range of operation, height of operation (above ground level), endurance, operating conditions, or level of autonomy, among others.

A highly extended criterion to establish a classification, is the lifting system, which distinguishes between: (1) lighter-than-air, (2) fixed-wing, (3) rotary-wing, (4) flapping-wing, and (5) hybrid configurations. Fixed-wing and rotary-wing configurations are the most widespread: while fixed-wing UAVs are mostly used on high-range, high-altitude, and long-endurance applications, requiring a runaway or launching/recovery system for take-off and landing, rotary-wing ones have the advantage of vertical take-off and landing (VTOL), hovering, and low-speed flights, at the cost of a lower range and endurance, which make them ideal for an even largest number of applications. Among the rotary-wing, the most common types are: (1) helicopter-type, and (2) multirotor-type. Helicopters come with higher payload and endurance with the disadvantage of being mechanically more complex, difficult to control, and dangerous (since they have larger propellers) than multirotor. For all the aforementioned reasons, multirotors are the most widely use UAV type in civilian and military applications, especially at flying heights lower than 120 m (see Section 3).

### 2.1. Sensors On-Board UAVs

UAVs are equipped with on-board sensors to acquire information on the environment (exteroceptive) or of the UAV itself (proprioceptive). Their use depends on many factors such as the environment, the application, and the tasks to be carried out; the payload capacity and size of the UAV; the cost of the UAV; safety and redundancy levels; the level of autonomy of the UAV, etc. Each sensor has different operational characteristics and therefore it comes with its own advantages and disadvantages. A whole review of the literature concerning all the different sensors that have been successfully used on-board UAVs is out of the scope of this paper. Thus, we will only introduce five sensor technologies that will appear later in the manuscript.

- *RGB cameras* are passive sensors that capture the intensity information of the visual spectrum of light of the observed scene, using three different channels, i.e., Red, Green, and Blue. Main issues when mounted on-board of the UAV are the vehicle speed or sudden burst that can cause blur and noise in the image. At this purpose, global shutter cameras, with the peculiarity that the entire area of the image is scanned simultaneously instead of a scanning across the scene (either vertically or horizontally) as for the rolling shutter counterpart, are usually preferred [23]. *Grayscale cameras* provide only one single channel with the average intensity information of the visual spectrum of light of the observed scene.
- *Event-based cameras*, e.g., Dynamic Vision Sensor (DVS) in particular, are well-suited for the analysis of motion in real time motion, presenting higher temporal resolution and sensitivity to light and low latency. Recently, it has been used on-board of UAVs [24]. Their output, anyway, is not composed by classic intensity imaging, but from a sequence of asynchronous events, thus the necessity for further research and the design of new image processing pipelines is very actual topic [25].
- *Thermal cameras* are passive sensors that capture the infrared radiation emitted by all objects with a temperature above absolute zero (thermal radiation). If their application fields were initially limited to surveillance and night vision for military purposes, their recent drop in price opened up a broad field of applications [26]. In fact, thermal imaging can eliminate the illumination problems of normal grayscale and RGB cameras, providing a precise solution in all the domains where life must be detected and tracked, and UAV does not represent an exception [27].
- *3D Cameras* can capture the scene providing color as well as 3D information. Presently, the three dominant technologies to build such cameras are stereo vision, time-of-flight (ToF) and structured light. For instance, commercial stereo cameras, i.e., the Stereolab ZED camera (https://www.stereolabs.com/), have been used for the UAV object detection problem [28]. Anyway, they are mostly based on triangulation, thus the presence of texture in the image is necessary to infer the depth information. With recent technological advancements, relatively small and low-cost off-the-shelf depth sensors based on structured light-like Microsoft Kinect ((https://developer.microsoft.com/en-us/windows/kinect/) [29]), ASUS Xtion Pro Live ((https://www.asus.com/us/3D-Sensor/Xtion_PRO_LIVE/) [30]) have been employed for object detection in UAV operations. Anyway, the aforementioned sensors are based on structured light, implying that they are sensitive to optical interference from the environment, thus more suited for indoor applications or controlled environments [31]. Finally, ToF cameras obtain the distance between the camera and the object, by measuring the time taken by projected infrared light to travel from the camera, bounce off the object surface, and return back to the sensor. They are becoming cheaper but are vulnerable to ambient light and UAV movements. Furthermore, they offer lower resolution than RGB sensors.
- *LiDARs* (Light Detection and Ranging), are active sensors that measure distances by hitting the target with laser light and measuring the reflection with a transducer. In former times, LiDARs were too large, heavy and costly to be used on-board UAVs, nevertheless, the recent advances on solid-state technologies, with examples as Ouster LiDARs (https://ouster.com/), have considerably reduced their size, weight, and cost, turning them into a growing popular choice on-board UAVs.

In most of the cases, *multi-sensor* solutions, where several sensors are used in a complementary way, are needed, to overcome the limitations of using a single sensor, or simply for redundancy. Multi-sensor fusion techniques focus on providing a robust and complete description of the environment or state of the UAV by combining observations coming from several different sensors [32,33].

RGB Cameras are the most prominent sensor to be used on-board UAVs due to: (1) reduced size and weight, (2) reduced cost, (3) reduced energy consumption, and (4) very large amount of data on their measurements. All these advantages come with the cost of a high-complexity of the information provided by their measurements (unlike other sensors, such as LiDARs that directly provide the distance to an object). As a consequence, RGB cameras represent by far the most employed sensors on-board UAVs. For this reason, in this survey, we focus on 2D object detection from RGB images. Nevertheless, we are aware that considering only RGB data would necessarily bring to illustrate an incomplete scenario. Thus, despite the main focus of this work will be the 2D case, useful references for further readings will be provided for the 3D cases as well as LiDAR, thermal imaging, and event-based cameras, giving priority to the cases of sensor fusion.

### 2.2. Autonomous Operation of UAVs: Situational Awareness

**Figure 1:**
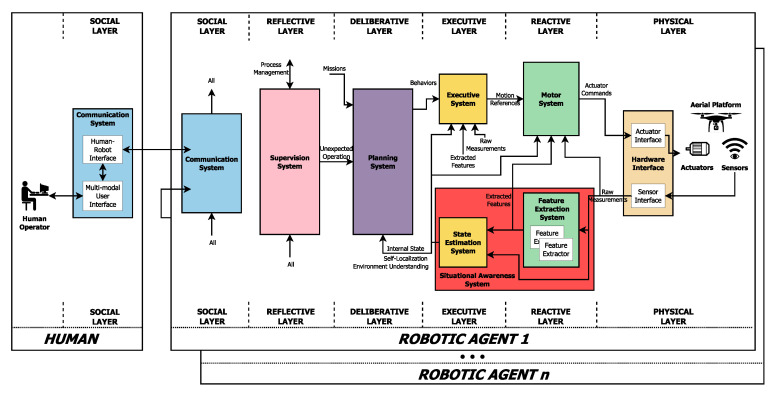
Aerostack system architecture [34]. The reader must note the importance of the situational awareness system (colored in red).

The fully autonomous operation of UAVs is needed to improve their performance, reliability, scalability, security, safety, ease its use, reduce its cost of operations, enable new applications, and to carry out applications based on 4Ds (Dull, Dirty, Dangerous, and Dear). Nevertheless, achieving a fully autonomous operation of UAVs remains an unsolved research problem. There exist multiple works focusing on versatile aerial robotics systems architectures to achieve such fully autonomous operation, as the successfully used Aerostack [34] (see Figure 1). Nevertheless, these architectures rely on the existence of several ready-to-use components with well-defined functionalities, that in most cases they have not yet reached the required level of maturity, performance, or functionality.

Among these components, one of the most important ones, and at the same time, the most challenging ones is the situation awareness. This component is in charge of the perception of the elements in the environment within a volume of time and space to generate a representation of the environment and the state of the UAV, by using the measurement acquired by the sensors (mostly the on-board ones) [35].

In many cases, the raw measurements provided by the sensors are too complex to be used directly in the situational awareness processes, and need to be simplified by extracting higher-level features. This pre-processing stage is carried out in feature extraction components [36]. The problem tackled on this survey, the 2D object detection, is an example of high-level feature extraction.

The importance of situational awareness lays in the fact that it is an essential input for the decision-making and control processes (see Figure 1). To carry out reasoning, these processes require the environment to be completely and accurately perceived, and often, their performance can be increased if the situation is modeled with different levels of information, such as metric, semantic or dynamic.

Semantic situational awareness intends to incorporate higher-level semantic information that augment the basic metric models of the environment, as for example [37]. Object detection provides a very valuable semantic information that can be exploited when building semantic maps, such as in [37,38,39,40,41,42].

**Figure 2:**
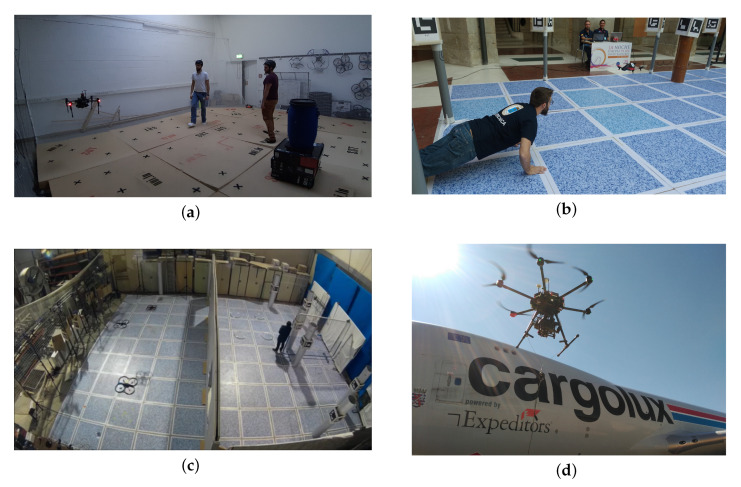
Examples of UAV applications, where situational awareness and object detection algorithms were used. (**a**) Obstacle avoidance using 2D LiDAR [42]. (**b**) Person following using RGB camera [46]. (**c**) Target detection using RGB camera [34]. (**d**) Airframe inspection using RGB camera, project FNR-PoC AFI, [53].

Examples of tasks and applications of UAVs that require situational awareness, where the use of an object detector is either essential or it substantially improves its performance (see Figure 2), are the following:

- Path planning or obstacle avoidance: consist on the generation of collision-free trajectories and motion commands based on the available knowledge of the existing obstacles of the environment [36]. Although it can be based on pure metric maps [43], its performance can be increased when having semantic information such as the kind of objects of the environment [44,45].
- Object or person tracking (following or servoing): consist on the generation of trajectories and motion commands that ensure that a particular targeted object (or person) are followed while they move [46,47].
- Exploration, survey or target detection and localization: consists on acquire information of the previously unknown environment, either to complete it, or to locate a particular element of it, e.g., [48,49].
- Surveillance, inspection or monitoring: consists on acquire information of the environment (normally previously known), looking for anomalies or intruders, e.g., [50,51,52].

## 3. Definitions and Proposed Taxonomy

The problem of 2D object detection from images is old as computer vision. It is very important to note that is no universal agreement in the literature of terms such as detection, localization, recognition, classification, categorization, verification, identification, annotation, labeling, and understanding, that are often defined in different ways [54]. In this work, the starting point is the nomenclature recently adopted in Liu et al. [55].

*Object detection* means to determine whether there are any instances of objects from given categories in an image and, if yes, to return the spatial location and extent for each object instance.

*Object classification* and *object categorization* refer instead to the problem of finding the presence of objects in the image from a given set of object classes, without any localization.

*Object recognition* is the problem of identifying and localizing all the objects present in an image [56], thus encompassing both image classification as well as object detection [57].

Furthermore, the problem of detecting a specific instance of a class object, e.g., “soda can” versus “coffee can”, or “my” mug versus “a” mug is referred in the literature as *object instance detection* [58].

Finally, these definitions can be easily extended for the 3D cases, with classification applied on 3D data (e.g., point clouds), but also in the case of 3D object position estimated directly from the 2D view. Thus, 3D object classification aims at classifying 3D data, while in the case of 3D object detection, the output is represented, in addition to the 2D bounding box, by the 3D position of the object(s), in real-world coordinates and in physical units.

Herein, we propose to classify the methods for achieving object detection depending on the flying height and the application domains that are unrolled at the different heights. Taking into account the computer vision point of view and integrating it in the existing taxonomic and categorization, we classify methods as:

- *Eye level view*: this category corresponds to a flying height between 0 and 5 m.
- *Low and medium heights*: 5–120 m. It represents the interval with the majority of commercial and industrial applications. Its upper bound has also been fixed according to the EU rules and procedures for the operation of unmanned aircraft (https://eur-lex.europa.eu/eli/reg_impl/2019/947/oj).
- *Aerial Imaging*: ≥120 m. It corresponds to elevated heights for whom usually special permissions are required to capture data, and/or satellite imaging.

The proposed ranges are motivated by the fact that eye level vision shares the viewpoint with many existing and well-investigated computer vision problems, although having new challenges when applying for the UAVs. Instead, once the UAV flies over a few meters, this classic viewpoint completely changes. Although the range extension is wide up to 120 m, such cases share the methodology and the state of the art network adaptation. Finally, there is another field concerning aerial imaging. These applications employ autonomous aircraft flying at a height starting from about 120 m up to kilometers; they also share different characteristics, i.e., pointing down cameras and specific datasets, thus, from the computer vision point of view, it is useful to consider this as a different category.

It is worth noting that these ranges are not strict, but are more related to the specific application context. For example, the eye level view mainly refers to the problem of UAVs sharing their trajectory with possible pedestrians and obstacles. Anyway, experimentally observing at existing works and datasets for urban navigation, it is possible to observe how the viewpoint is practically frontal usually up to 5 m of height; thus, these works should still considered in the category of eye level. On the other hand, works dealing with indoor navigation could theoretically fly at higher heights, but the challenges involved in the works herein analyzed deal with cluttered environments and the presence of obstacles, i.e., situations that appears in human height navigation, thus the methodology is still valid in the case of a slightly higher height, and we grouped them together.

Finally, we are aware that changes in viewpoint, angulation, sensor suite and light conditions can noticeably change the approach even at the same fixed height, as well as that the same object class can be detected from different heights. Anyway, we think that this representation is very useful to map the different architectures and themes that are specific from each specific application, as well as to classify the possibility of extending the functionalities in terms of multi-modal sensor fusion schemes.

## 4. Object Detection Architectures

Early object detection algorithms were based on a handcrafted features extraction phase upstream, searching for sophisticated feature representations that capture the gist of the image. In those years, a variety of tunes-ups to improve accuracy, obtaining invariance with respect to various geometrical and spectral factors, and (near) real-time performance were proposed in the literature [59,60,61], reaching a plateau after 2010 [62]. In 2012, the seminal work of Krizhevsky et al. [63] gave the rebirth of Convolutional Neural Networks (CNNs) for the image classification task. *AlexNet* (2012) consists of 5 convolutional layers and 3 fully connected layers. Two Graphics Processing Units (GPUs) were used, running different layers of the network and communication only at certain layers. The paper introduced modern concepts such as data augmentation, training on multiple GPUs, Rectified Linear Unit (ReLu) as activation function, max-pooling layers to achieve downsampling, drop-out as a way to perform regularization, achieving a top-5 error of 15.3% on ImageNet Large Scale Visual Recognition Challenge [64]. From that moment, different architectures have been proposed to improve the accuracy of such a task [55,62]. Here, we report works that represent a milestone achievement or that will be recurrently named in the manuscript:

- *R-CNN* [65] (2014) is a two-stage object detector that introduced the search for possible object locations (region proposals) in the image using a selective search; for each proposal, the features were extracted separately by the network, at the cost of high computational load.
- *Faster R-CNN* [66] (2015) increased speed of R-CNN taking the entire image as input instead of using a CNN for each region proposals introducing RoI pooling layers, improving both accuracy and speed.
- *Mask R-CNN* [67] (2017) extended Faster R-CNN, adding a branch for predicting also the object mask in parallel with the bounding box recognition.
- *YOLO* [68] (2016) is a one-step object detector that drastically improving computing speed. Like Faster R-CNN, YOLO uses a single feature map to detect objects, but the image is divided into a grid where the objects are searched. From the first version of YOLO, many further improvements have been proposed, leading to different YOLO versions [69].
- *Feature Pyramid Network* (FPN) [70] (2017) has turned out to be fundamental for the correct identification of objects at different scales. This concept has been proposed numerous times along with light or more radical modifications since its publication so that it can be considered to be a structural component of modern object detectors. Pointedly, in the original FPN version, a top-down path is linked with lateral connections with the usual bottom-up feed-forward pathway to output multi-scale prediction in correspondence to the multi-resolution feature maps.

For our purposes, instead, it is important to highlight how neural networks specifically designed for mobile devices gained tremendous importance. This section is concluded by milestone works that have been designed for performing inference on low power and memory devices. Firstly, efficient architectural designs are described. Lastly, techniques to further improve run-time performance while preserving accuracy are introduced.

- *SqueezeNet* [71] introduces a new building block, called the *fire module*, which is composed by a 1×1 convolutional layer to reduce the number of channels with the minimum number of parameters (hence the name *squeeze*) followed by a mix of 1×1 and 3×3 convolutions forming the *expand* block that increases again the depth dimension. Additionally, this module is combined with the design decision of delayed down-sampling, by which the feature maps resolution is decreased later in the network to improve accuracy.
- *SqueezeNext* represents an upgrade of the aforementioned network [72]: the authors reduce the depth to a quarter by using two bottleneck layers. Furthermore, through a separable convolution, they swipe over the width and the height of the feature maps in other two consecutive layers before expanding again the channels with a 1×1 convolution. Finally, a skip connection is added to learn a residual with respect to the initial values.
- *ShuffleNet* [73] makes use of group convolution in the bottleneck layer to reduce the number of parameters. Noticing that the output would depend only on one group of input channels group convolutions, a mechanism to break this relation is under the form of a shuffling operation, which is feasible by simply rearranging the order of the output tensor dimensions. Nevertheless,
- Ma et al. [74] argue that the memory access cost of the group convolution would surpass the benefits of the reduced floating points operations (FLOPS) version, and consequently, proposed a second version of the shufflenet architecture based on empirically established guidelines to reduce computation time. In this regard, with *ShuffleNetV2*, the authors introduce the *channel split* operator that branches the initial channels in two equal parts, one of which remains as identity and is concatenated at the end to restore the total number of channels. Hence, the 1×1 group convolutions are substituted with normal point-wise convolutions and the channel shuffle operation of the original model is replicated after the concatenation of the two branches to allow information exchange.
- *MobileNet* [75] is built upon the concept of *depthwise separable convolutions*, first introduced by [76] and popularised by the Xception network [77]. This computation module consists of one first layer of kernels that operates on each input channel independently, the depth-wise convolution, followed by a second, the point-wise, that combines the intermediate feature maps through a 1×1 convolution. Additionally, MobileNet has a hyperparameter, named *width multiplier*, to control the number of output channel at the end of each block and the total number of parameters.
- *MobileNetV2* [78] adds linear bottleneck layers at the end of the separable convolutions to create what the authors call *inverted residual block* since the skip connection with the identity feature maps is performed when the network shrinks the number of channels. The intuition of the authors is that ReLU non-linearity can preserve the information manifold contained in the channels when it lies in a low-dimensional subspace of the input.
- *Neural Architecture Search* (NAS) is a novel prolific field whose purpose is to use search algorithms, usually either Reinforcement Learning or Evolutionary optimization methods, to find a combination of modules that obtains an optimal trade-off between latency and accuracy. For instance, the optimization metric could be specified in the form of a Pareto multi-objective function as demonstrated in *MnasNet* [79]. *FBNet* [80], *ChamNet* [81], and *MobilenetV3* [82] are other examples of successive contributions to mobile network architecture search. The reader can refer to [83,84] for a thorough analysis of this technique.
- *EfficientNet* [85] improves the ability of manually tuning the model complexity letting the users choose the desired trade-off between efficiency and accuracy. Pointedly, the authors propose a *compound scaling* hyperparameter that tunes the network depth, i.e., the number of layers or blocks, the width, i.e., the number of channels, and the input image size, which influences the inner feature maps’ resolution, all at once in an optimal way. This scaling method follows from the observation that the network’s dimensions are not independently influencing the latency-accuracy trade-off. Finally, this novel scaling concept is applied to a baseline network found by taking advantage of the multi-objective optimization and search space specified in MnasNet.

In [86,87] exhaustive overviews regarding the techniques for model compression and latency reduction are provided. Herein, we describe the principal characteristics of those methodologies.

- *Parameter Pruning* is a technique principally meant to reduce the memory footprint of a neural network by reducing the redundant connections. In general, single weights, group of neurons, or whole convolutional filter can be removed improving also the inference time. As a side-effect, this operation impacts the accuracy of the model, thus requiring an iterative process composed of pruning and network re-tuning steps. Additionally, the resulting models are generally sparse and require specialized hardware and software to not lose the advantage over the unpruned dense counterparts.
- *Quantization* cuts the number of multiply-adds operations and the memory storage by changing the number of bits used to represent weights. Since 16, 8, or 4 bits are more commonly used, for the special case of 1-bit quantization we refer to weight binarization. Additionally, weight sharing is a related concept that indicates the technique of clustering groups of weights that fall into an interval and to assign them a single common value.
- *Knowledge Distillation* has the objective of transferring the knowledge embodied into large state-of-the-art models to lighter networks, which would have the advantage of retaining the generalization capabilities while being faster. This technique can be coupled with quantization where a “teacher” network would use full-precision representation for the weights and the lighter “student” network is, for example, binarized. However, applying this method to tasks other than image classification, where the compressed model learns to mimic the class logits from the teacher, is more challenging. In this regard, the work in Chen et al. [88] shows how to generalize this technique to object detection by exploiting the teacher regression loss relative to the bounding boxes’ coordinates as an upper bound.
- *Low-rank Factorization* replaces weight matrices with a smaller dimensional version using matrix factorization algorithms. For instance, *Singular Value Decomposition* can be applied to perform a low-rank factorization that approximate the original model parameters by retaining the top *k* eigenvalues of the weight matrix.

## 5. Eye Level View

This section introduces to the works in the state of the art that focus on drones flying at men height. Generally speaking, flying at such height means that the same viewpoint, variance and appearance of many classic computer vision use cases are shared. Anyway, own specific challenges are present, from both computational and scientific point of view.

Due to the limited computational capabilities on-board, many works tend to employ classic computer vision techniques in order to achieve a higher frame rate. Real-time obstacle detection and avoidance system based on monocular views has been proposed in [89]. Features of the obstacle are detected with SIFT, and keypoints matching with respect to a stored patch is performed with a brute-force approach. Ratio and distance filters are applied to obtain a more robust match. The convex hull is used to establish the object of interest that must be avoided, and changes in the size of the area of the approaching obstacle to performing the avoidance manoeuvre are used to estimate the obstacle distance. More recently, in [53] the problem of airframe visual inspection from UAV monocular imaging has been addressed computing the Features from Accelerated Segment Test (FAST) and Oriented Rotated Brief (ORB) descriptors to find features that are matched to detect if one landmark (among a set of known landmarks) is in the scene, and to estimate the airplane pose with respect to the flying drone in real time. Apart from the feature extractor phase, this approach presents two main differences: first of all, matching is performed by using the fast K-Nearest Neighbor search relying on a multi-probe Locality-Sensitive Hashing (LSH) [90]. The classic LSH is computationally lighter than the brute-force approach. In turn, the multi-probe LHS is still based on the classic LSH, but more efficient indexing is obtained since it probes multiple buckets that are likely to contain query results in a hash table. Moreover, the object is recognized in terms of 2D coordinates, but the distance is estimated by the iterative PnP algorithm based on Levenberg-Marquardt optimization [91].

In [92], a combination of features with Adaboost classifier has been used to detect pedestrians. In particular, a combination of Haar-like features and Local Binary Pattern is employed, and the mean shift is introduced to improve the performance in a flight environment between 2 and 4 m. Differently from the previous two works, a training phase is required. The *CICTE-PeopleDetection* dataset is also introduced, and images at heights lower than 5 m have been considered for the training and test sets. Finally, to improve the classification performance, a mean-shift algorithm is introduced to track the pedestrians. In the experiments, authors show how this solution improves the performance at elevated heights, when the view-point angle increases, but at the cost of introducing more false positives.

Real-time detection and tracking of pedestrians fully performed on-board of the UAV has been proposed in [93]. A detector based on Aggregate Channel Feature (ACF) is employed and combined with ground plane estimation to reduce the search space, based on the sensors data of the UAV. This leads to an improvement in terms of efficiency and accuracy. The system has been integrated with a particle filter based on color information to track the pedestrians and, in turn, the pedestrian detector helps the particle filter to remain on target. In the experimental phase, a pedestrian following scenario was also presented.

It is worth mentioning that in other cases [94,95] the obstacle is modeled implicitly. In particular, visual information is taken from the on-board camera(s) and used for guidance, implementing an obstacle avoidance solution based on optical flow variations.

From the other side, recent advances in deep learning represent a unique opportunity to improve the capabilities of providing scene awareness for a UAV operating in realistic environments. From the analysis of the state of the art, it emerges that application of deep learning techniques from eye level heights can occur in two ways: one solution, certainly interesting from the engineering point of view, is to simply switch the computation off-board, e.g., using cloud computing. For this purpose, the authors in [96] applied a resource-demanding neural network, i.e., R-CNN, in a consumer UAV (a Parrot AR Drone 2.0) to detect hundreds of object types in near real-time. The second way, very recent and that has maybe more appeal from the scientific point of view, is to develop ad-hoc architectures and/or to adapt the existing backbone networks.

An example is the work in [97], where on-board and real-time trail detection and following has been implemented for a micro air vehicle flying in a forest. The backbone of the proposed CNN architecture (named TrailNet) is ResNet-18 [98]; moreover, a modified version of YOLO is used to detect objects (e.g., persons) and avoid collisions.

Finally, it is worth mentioning machine learning approaches that directly maps visual input with actions. This research field is not new [99], but recent advancements in deep learning have brought unseen performance and innovative approaches to implement biologically-inspired controllers. An example is in [100], where a CNN that directly predicts the steering angle and the collision probability from RGB images has been proposed. The network was trained with data recorded from ground vehicles and the predicted output is used to control the UAV to safely navigate avoiding obstacles. In this way, the obstacle is implicitly modeled by the collision probability that is 0 when the object is far away and becomes 1 when the collision is already unavoidable. Moreover, authors report how activation maps show the focus of the network on objects such as street lines that are an indicator of steering directions. Differently from deep learning-based control approaches that focus on reinforcement learning schemes, this family of works relies still on supervised learning, and it represents a very active research field [101].

### 5.1. Human-Drone Interaction

A drone flying at human height ineluctably interfaces with persons. Human-drone interaction (HDI) represents a wider and multidisciplinary research area that has spread in the last ten years, involving computer vision and robotics but also social sciences and human-computer interaction, with research involving proxemics [102], attention schemes [103], design [104], social perception [105], drone personality definition [106].

In this field, computer vision becomes fundamental to realize an interaction that can take place in realistic scenarios, recognizing the humans, but also their body parts and gestures [107]. In [108], Pose-based Convolutional Neural Network (P-CNN) descriptors [109] have been employed on the UAV-GESTURE dataset to recognize gestures with an accuracy of 91.9%. A similar approach has been used from the same authors on the Drone-Action dataset, released one year after, obtaining an accuracy of 75.92%.

**Figure 3:**
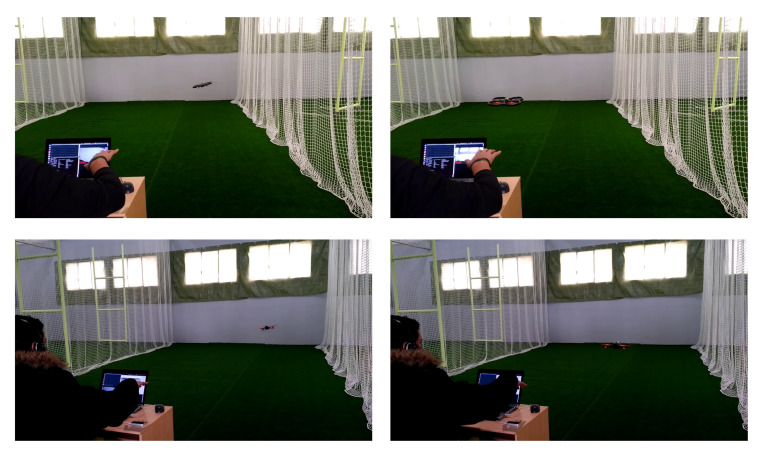
Four examples of human-drone interaction performed by hand gestures in [110].

In the work of [110], a natural user interface to interact with drones has been designed and tested. Among others, the UAV can recognize a person that can control the drone using body pose and hand gesture. In particular, hand movements are tracked by using a Leap Motion Sensor (https://www.ultraleap.com/product/leap-motion-controller/), a device specifically designed to track hands. It consists of two webcams with fish-eye lenses, three infrared LEDs and a plastic diffusing panel. A ground station (i.e., a laptop communicating in the same network of the UAV) can transmit commands to a Parrot AirDrone 2.0. The body is tracked with the Aerostack framework. In the same work, also visual markers are used to transmit commands. Some output of the gesture recognition module is reported in Figure 3. The face has been used to control the robot in [111]. The user triggers the robot to fly by making a distinct facial expression, and the direction and intensity of flight are given by the head position and size; three different trajectories have been implemented and tested. The orientation of the hand regarding the head pose (discretized into three possible poses) is used to estimate the direction of the UAV flight in [112]. An attempt to interact with a swarm of flying robots using hand gestures has been provided in [113]. Gestures are also used for selecting individuals and groups, using a vocabulary of two-handed spatial pointing gestures. Generally speaking, many works that exploit gestures to control a single drone have been proposed in the literature [114,115], showing a robust and mature technology. Similarly, real-time facial detection and recognition from UAVs represent two well-investigated problems [116,117]. A study about limitations and the impact of different viewpoints and heights for the recognition task has been also proposed in [118].

### 5.2. Indoor Navigation

As light and small sensors with high precision and accuracy performance and low latency emerged, many navigation architectures for indoor flights have also been proposed. A summary of UAV indoor navigation architectures can be found in [119]. SURF features for template matching to compare relative obstacle sizes with different image spacing have been employed to detect and avoid obstacles during indoor test flights in [120]. The detection of objects such as studs, insulation, electrical outlets, drywall sheets (for the latter, also its status is classified) in indoor construction sites has been proposed in the work in [121]. One of the validation cases has been a dataset of images collected by a UAV, with promising performance for the autonomous inspection task.

CNNs have been applied to identify waypoints in dynamic indoor environments for drone racing [122]. Waypoints are identified in local-body frame coordinates to deal with the problem of drift, and are integrated with state of the art path planner and tracker. A big innovation introduced with this paper is that the network is trained in simulation and deployed on a physical quadrotor without any fine-tuning, representing the first zero-shot sim-to-real transfer on the task of agile drone flight.

Finally, also event-based cameras have been applied for improving autonomous indoor navigation, where the continuous presence of obstacles and/or walls provide significant input. For example, in [24], they are used to create an event stream representation that uses the temporal component of the stream, whose 3D geometry is approximated with a parametric model to compensate the camera motion; in this context, moving objects not conform with the model are detected and tracked, even in the case of very fast motion. In this work, the object detection is implicit since achieved with a different motion model with respect to the moving UAV.

### 5.3. Datasets

*CICTE-PeopleDetection* [92] aims at detecting pedestrians in the scene. The authors state that existing related datasets are not considering viewpoints changes that occur in the case of UAVs operating at eye level heights. Thus, they introduce a dataset of pedestrian images composed of 30,900 positive (with one or more pedestrian) and 12,120 negative (no pedestrians) samples. UAVs behavior is emulating changing the height of surveillance cameras, from 2.3 to 5 m, with a resolution of 50×100 pixels.

*DroneFace* [123] has been designed for face recognition purposes. Ages range from 23 to 36 years old, and heights from 157 to 183 cm. All the subjects have been asked to take frontal and side portrait images before the raw image collection. The dataset is composed of 2057 images at 3680×2760 resolution, but the UAV mobility is only simulated.

The first large-scale dataset of images collected from a UAV with the aim of testing face recognition performance is *DroneSURF* [124]. The dataset contains 200 videos of 58 different subjects, captured across 411,000 frames, with over 786,000 face annotations. Videos have been captured using a DJI Phantom with a resolution of 720 p and 30 fps. Heights range has not been given by the authors, but sequences show variability at eye level and very low heights.

*UAV-GESTURE* [108] is a dataset containing 13 gestures for basic UAV navigation and command from general aircraft handling and helicopter handling signals. It is composed of 119 high-definition video clips consisting of 37,151 frames recorded by a hovering UAV.

*Drone-Action* [125] is recorded instead from a flying UAV, recording 13 human actions, for a total of 240 videos and 66,919 frames. The actions were recorded in three different settings (following, side-view, and front-view actions). Examples of recorded actions are clapping, punching, hitting with a bottle, walking, running, allowing the testing of people monitoring systems.

*DroNet (ETH)* [100] is the dataset used in the work of the same authors to estimate the collision probability and the steering angle to avoid it. It is based on images taken from the Udacity dataset (https://github.com/udacity/self-driving-car) that has 70,000 images together with the information from a complete sensor suite. Here, only the steering angle is taken. Concerning the probability prediction of a collision with an obstacle, 32,000 images have been collected and manually labelled with a probability between 0 and 1. The authors started to record when far away from an obstacle (p=0) and stop when very close to it (p=1). 137 sequences to model a diverse set of obstacles have been created.

## 6. Low and Medium Heights

This section gathers works that apply computer vision and, particularly, object detection techniques to aerial vehicles that are expected to fly in that band of air space between 5 and 120 m. This flight zone is the most natural for the majority of commercial applications dealing with UAVs and, consequently, attracts most of the research community focus. Because of the versatility achievable with a drone capable of detecting objects in such an extended range of heights, we tend to consider this category rich of challenges to work out. Notably, the main difference with the other two flying heights categories of the proposed methodology classification is the high variance in perspective and size with which objects can appear from the cameras mounted on the drones.

In respect of these observations, and considering the impact on computer vision deep learning brought in the last decade [126], the most recent solutions to overcome the challenges involved in object detection from an aerial point of view primarily revolves around the use of CNNs. Nonetheless, in practical applications (e.g., Section 6.1 and Section 6.2), the adoption of more classical and engineered computer vision pipelines is not rare, especially given the lack of adequate computing resources, such as embedded GPU, or caused by the absence of labeled data for a specific task/environment.

To address the scale variability issue in aerial images, different neural network modules have been proposed. In particular, Yang et al. [127] observe that the detection targets are in general small compared to the high resolution of the images and that these are usually sparse, non-uniformly distributed, and clustered. Therefore, they propose a three-step pipeline composed by specialized sub-networks: the first, CPNet, similarly to a region proposal network, extracts candidate clusters merging the regions where target presence is denser; secondly, ScaleNet estimates the scale of the objects’ bounding boxes contained in each cluster; finally, the input is rescaled and padded accordingly to the previous step for feeding into a standard detection network, DetectNet, the cluster regions separately. Similar observations regarding the distribution of objects in aerial images led Li et al. [128] to introduce the concept of density map for performing the detection on cropped image regions. The results are then fused with the object detection results on the entire image followed by a non-maximum suppression post-processing step to merge overlapping boxes. Furthermore, in [129] a Generative Adversarial Network trained for Super-Resolution, SRGAN [130], is applied to up-sample the extracted crops based on a learned Normalized Average Object Relative Scale factor (NAORS) in conjunction with the more common bi-linear interpolation.

Notwithstanding the importance of the detector accuracy on objects with variable size and viewpoint, another compelling aspect for UAVs’ software development is that of real-time performance. To this extent, SlimYOLOv3 [131] is the result of channel-wise parameter pruning applied to the popular YOLO object detector imposing an L1 regularization loss on the learned batch normalization statistics. However, the authors show that even though the reduction in parameter gives an advantage over the vanilla YOLO implementation, the shallower architecture of YOLOv3-TINY still obtains higher FPS claiming that a bottleneck in the pruning does not allow further improving the performance. With a different approach toward network efficiency, the work in [132] introduced Mixed YOLOv3-LITE, a lightweight architecture for real-time performance. The network is based on YOLO-LITE [133], but residual blocks, as well as an implementation of parallel high-to-low resolution sub-networks, are introduced, achieving an optimal balance between performance and speed even in non-GPU-based computers or portable terminal devices. Testing in both eye level and low-medium heights settings, the architecture achieved 43fps with an input of 224×224 pixels and 13 fps with 832×832 pixel images.

Finally, in [134], a multimodal sensor fusion scheme to improve UAV-based image classification performance has been proposed. In particular, two sensor fusion configurations are compared: the first one consists of a Panchromatic and color camera, the second one of a four-band multi-spectral camera. The resulting images are compared to the ones acquired by a high resolution single Bayer-pattern color camera. Experiments show how both sensor fusion configurations can achieve higher accuracy compared to the images of the single Bayer-pattern color camera. Also, a 2D LiDAR with a stereo camera for safe navigation in an autonomous flight of a multi-copter UAV has been implemented [135], showing that the research is giving a lot of attention to this promising area.

Among the applications fields, search and rescue and surveillance are arguably the ones that would benefit the most from the support of a drone for detecting people from high above the ground, specifically in the range of heights that the proposed taxonomy names as low-medium. Other tasks, for example, precision agriculture or building inspections, require detection of objects too tightly tied to the use case under examination, but a generalization from the concepts and techniques depicted herewith is possible. In light of this, the remainder of the section focuses on these prolific research areas.

### 6.1. Search and Rescue

As aforementioned, an application for which drones generally operate between 10 and 100 m of flight height is Search and Rescue (SAR). Whereas for an Emergency Medical Technician (EMTs) reaching the victims in areas where a natural disaster has occurred could be difficult and dangerous, it is faster and safer when these actors are aided by robots that can overfly an area to check in advance the location of those in needs of their assistance. Therefore, among the different challenges, the detection of distressed people and other objects of interest for the support mission is a central problem to solve for obtaining an autonomous system for supporting life-guarding teams. Nevertheless, the design of such systems varies accordingly to characteristics of the environment in which it is supposed to operate.

Urban Search and Rescue (USAR)’s general requirements are described by Półka et al. [136], who are committed to their project, called MOBNET, aimed to support teams with a system capable of localizing isolated victims of natural disasters. In this regard, they surveyed 300 potential end-users with questions about the technical capabilities that should be included during the system design. In [137] a system for the coordination of first responder rescue teams in emergencies, such as firefighters, is imagined with the assistance of a UAV equipped with sensors and embedded computers. To generate alerts, the aerial robot should detect dangerous objects to warn preemptively the human operators. The correct selection of the object detection algorithm in terms of a trade-off between speed and accuracy is vital for such delicate tasks. Hence, Tijtgat et al. [137] compare two methods, i.e., Aggregated Channel Features (ACF) [138] and YOLOv2 [139], which are antithetic due to the nature of their approaches. Whereas the first makes use of engineered features, i.e., histogram of oriented gradients, coupled with an ensemble of decision trees, i.e., AdaBoost [140], the second applies a CNN for extracting features and regressing bounding boxes coordinates. Both methods are implemented having in mind maximizing the use of the GPU, since an NVIDIA Jetson TX2 is thought of as the main computing platform on the drone. As a result, YOLO reveals to have a better Precision to Frame Rate ratio with respect to ACF by virtue of its additional compatibility with the GPU parallel nature. In light of this, the widespread introduction of consumer-grade embedded GPUs, e.g., the popular NVIDIA Jetson TX1 and TX2, made it possible to easily transfer the knowledge regarding neural networks from cloud-computing to mobile robotics and, eventually, to level up the solutions for object detection.

With regards to rescue missions in an aquatic environment, ROLFER [141] is an autonomous aerial rescue system (AARS) designed to intervene in life-threatening situations, for example, drowning swimmers. It is composed of an UAV capable of autonomously navigate towards a location inside the *supervised area* within which a user sends a distress signal that remotely activates the assistance mission. Concerning the navigation aspect, a base control station is in charge of the mission planning by sending control commands to the UAV. Additionally, the station relies on the GNSS positioning system for the localization of the swimmer and the flying drone itself. Instead, the detection of the swimmers is performed in real time on board of the UAV using deep learning techniques. However, the training of a neural network requires images specific to the task, which is not always openly available. For this reason, Lygouras et al. [142] released the Swimmers dataset that contains 9000 images of annotated swimmers at different angles and lighting conditions taken from a drone flying over the water. Training a state-of-the-art deep object detector, i.e., TinyYOLOv3, with a multi-scale strategy, the authors reports a 67% mAP (mean Average Precision) and 70% recall as a baseline for future benchmarks. Furthermore, they argue that the autonomy gained using a light network is fundamental in time-critical missions, which would not allow a delay for transmitting images back and forth to a more powerful server.

Opposite to urban scenarios, the challenge of search and rescue in wild environments (WiSAR) implies to fly over extended regions of forests or deserts with the consequence of requiring extended flight-times. Hence, Sun et al. [143] build a system for autonomous long time missions upon a fixed-wing airframe and an action camera flying above 80 m. Instead, regarding the on-board target detection, this is the result of a simple pipeline, which transforms the aerial images in the YUV space and then identify color signatures by thresholding. As a result, the identification range is restricted and requires that the victims wear particular clothes to be detected. To endure long flight times, in [144] an RPH-2 UAV, a mini helicopter with an hour of endurance a 100 kg of maximum payload, is adopted to conduct experimental missions on a riverside. A camera with a large focal length pointing down and a with a front-facing video camera completes the photography equipment. Similarly, in [145,146] a Yamaha RMAX helicopter is customized to find survivors in disaster areas. Notably, besides an RGB sensor, the human body detection algorithm can rely on thermal camera views, which is calibrated with the color images to find correct pixel correspondences. In this way, during the mission, the thermal image can be processed to find candidate regions that present a plausible human body temperature and further filtered with a body shape heuristic. Finally, the Viola and Jones detector [59] is used to identifying the presence of people in the registered color image. Therefore, the coupling of the thermal imagery with classical object detection techniques, which uses inefficient region proposal pipelines, allows pruning from the image-region search space those areas not consistent with a priori knowledge of the human body.

### 6.2. Crowd Analysis and Monitoring

Visual crowd surveillance and analysis from cameras mounted on the UAV can provide safety to events gathering many people by recognizing suspicious human activities or criminals, in real-time and in an efficient manner [147]. In aerial robotics, advanced communication systems, e.g., LTE 4G and 5G mobile networks, can be exploited to support the long-distance, elevated height, and high mobility characteristic nature of UAVs [148]. Transmitting data to high-performance computing infrastructure, or exchanging data with IoT devices on the ground, enables the support of external sources of information in the case of multi-sensor systems and multi-agents operations [149].

**Figure 4:**
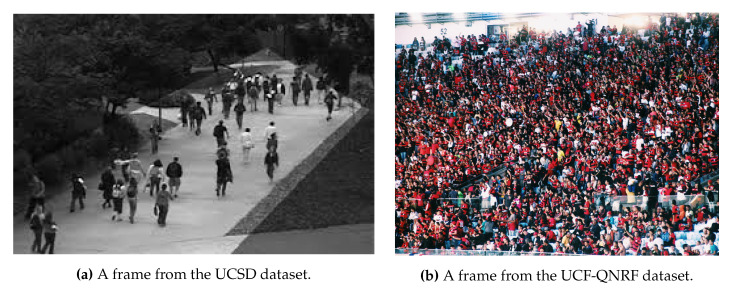
Two examples of crowd analysis images.

People counting is an attractive task as it is lately emerging as a new frontier in computer vision and as it is giving an extended operative application to the UAVs. Since people counting applies on a wide range of scenarios, from low-dense to extremely crowded scenes, two example images that illustrates the difficulty scale is given in Figure 4. Relatively to this task, the lack of training samples, occlusions, cluttered scenes, complex backgrounds, non-uniform distributions, and perspective variations embody the main challenges researchers have to face. In this scenario, state of the art methods can do a regression, i.e., directly map the image with the number of people [150,151], a detection, i.e., localizing each person composing the crowd, or a density estimation, grouping the regression and the localization tasks. The last two categories are attempts to address this problem as a real object detection instance, and thus some seminal articles will be provided in this section.

FAST algorithm has been used to detect the crowd features from UAV images in [152]. In particular, a circle of 16 pixels surrounding the center pixel is converted into a vector and sorted in ascending/descending order. FAST-9 and FAST-12 are used and compared in representing crowd features, that are used to count people. A model merging SIFT descriptor and different texture analysis methods like Fourier analysis, GLCM features, and wavelet transform have been proposed in [153]. CNNs have been employed as a sparse head detector in dense crowds in [154]. The image is divided into rectangular patches, and SURF features are fed to an SVM binary classifier to eliminate non-crowded patches. For each patch, an estimation of the number of individuals is given by counting heads or using on distance-based weighted averaging if no heads are present. Finally, the individual patch counts are summed up to obtain the total count. In the work of [155], a scale driven convolutional neural network (SD-CNN) model is adopted, based on the consideration that heads are the dominant and visible features regardless of the density of crowds. Because of the scale problem, a scale map representing the mapping of head sizes is created, and scale aware proposals based on the scale map which are extracted are fed to the SD-CNN model acting as a head detector. At last, non-maximum suppression returns the final heads’ positions. An example of density-based estimation is in [156]: an encoder-decoder architecture composed of inception modules is used to learn the multi-scale feature representations. A multi-loss setting over different resolutions of density maps is used to adapt to image resolution. Multi-task learning learns the joint features for the density map estimation task and the density level classification task. Lastly, the U-net architecture is introduced so that the encoder and decoder features are fused to generate high-resolution density maps. With DensePeds [157], people in dense crowds are detected, and positions are tracked over time in both front-facing and elevated views. A motion model called Front-RVO (FRVO) is introduced to predict pedestrian movements using collision avoidance constraints; this component is combined with Mask R-CNN to compute sparse feature vectors that reduce the loss of pedestrian tracks. Counting, density map estimation, and localization from a single CNN with a decomposable loss function have been proposed in [158].

A real-time autonomous UAV-based surveillance system to identify violent individuals in public areas from flight heights between 2 and 8 m has been introduced in [159]. A feature pyramid network (FPN) is used to detect people in the image, whose pose is estimated by a ScatterNet Hybrid Deep Learning (SHDL) network, introduced by the authors. Real-time performance is achieved by processing images in the cloud. People analysis has been provided by recognizing actions in [160]. A two-stream CNN architecture coupled with a temporal pooling scheme called to produces nonlinear feature subspace representations have been tested with the MOD20 dataset.

Generally speaking, this problem has received particular attention from the scientific community, with specific challenges and review papers. Refer to [161,162] for a discussion on the CNN impact on the crowd behavior analysis.

### 6.3. Datasets

*UAV123* [163] is a dataset devoted specifically to tracking objects releasing a set of 123 HD annotated video sequences. An assorted range of targets, e.g., from pedestrians to different types of vehicles, are represented in various environments such as urban landscaped, sea coasts, industrial areas, and agriculture fields. Additionally, a small set of videos is generated using the UnrealEngine4 simulation engine that features high-quality renderings of real-world scenarios (https://www.unrealengine.com/en-US/industry/training-simulation). As a motivation for benchmarking on virtual scenarios, the authors argue that an accurate flight physics simulation carries the advantage of integrating the tracking into the navigation controller loop.

*VisDrone* Detection Challenge ’18 [164] and ’19 [165] provide a collection of 8599 images captured from a drone in different urban scenarios and varying the height of flights. In particular, the dataset has been collected using different drones flying to fly over numerous cities experiencing variable weather conditions. Each image contains annotations of various objects belonging to one of ten categories reaching a total of 540 k bounding boxes. The authors provide specific tracks for single and multiple object tracking challenges and a few videos of low height flights following targets [166]. Besides the bounding box coordinates and object class labels, the authors provide the occlusion ratio and truncation ratio as additional annotations. More recently, the benchmark has been extended with a crowd counting challenge (http://aiskyeye.com/challenge/crowd-counting/) proposing what is probably the first dataset in his kind to collect images from a UAV.

*UAVDT* [167] is a benchmark to test the tasks of object detection and tracking in the environmental conditions typical of UAVs, that is, crowded objects due to wider viewing angles, small sizes, and camera motion. Additionally, the images were captured under different weather conditions (daylight, night, and fog), at different heights (from 10 to more than 70 m), and consisting of three camera orientations allowing multiple views of the objects (front, side, and bird’s-eye views).

The *Swimmers* dataset was published in [142] specifically for maritime search and rescue missions. Due to the lack of visual characteristics represented by people drowning or floating in the water in the most popular datasets, it provides a valid alternative for people detection in this particular environment. The dataset is limited to about 9000 images captures from a drone with a GoPro camera in full HD resolution and mixed with some others collected from the Internet.

The *Stanford Campus* [168] dataset’s purpose is to model socially influenced trajectories of people walking inside the Stanford university campus. To this end, additional to people and some vehicle bounding boxes, annotations representing *target-target* and *target-space* interactions are provided. The observation of the authors is that humans follow various social principles when interacting with other people and or particular environment characteristics, e.g., following road paths, leading to the definition of *social sensitivity*, the level of target interaction along a trajectory.

With an emphasis on traffic surveillance, the *AU-AIR* [169] dataset propose 32,823 labeled video frames captures during low-level flight over road junctions. In particular, bounding boxes of eight objects categories, e.g., person, car, bus, van, truck, bike, motorbike, and trailer, have been manually annotated. Notably, this is the first dataset for UAV object detection to include additional sensor data logs, such as altitude, IMU, and GPS making multi-modal learning or even to benchmarking other tasks related to surveillance, such as localization, an additional possibility.

Concerning people counting, the majority of the datasets are composed of a few hours of people monitoring in a static scenario. For example, *UCSD* [170] dataset contains one hour of low-density crowd collected from a stationary digital camcorder overlooking a pedestrian walkway at UCSD. The video is resized to 238×158 pixel resolution and sampled to 10 FPS. Also, the traveling direction and visible center of each pedestrian are annotated every five frames, while pedestrian locations in the remaining frames are estimated with linear interpolation. An attempt to provide a large scale dataset showing different conditions and very crowded scenarios is the *WorldExpo’10* [151], with 1132 annotated video sequences captured by 108 surveillance cameras during the Shanghai 2010 WorldExpo. A total of 199,923 pedestrians have been labeled. Also, the *UCF-QNRF* dataset [158] overcomes the shortcomings of previous datasets, introducing 1.25 million humans, manually marked with dot annotations in 1535 images.

Recently, *MOD20* propose a hybrid dataset which integrates YouTube videos and UAV/aerial images [160]. The dataset provides 2,324 video clips for a total of 503,086 frames at a resolution of 720×720. Such a task is incredibly promising as a testbed for human action recognition in the wild from both ground and aerial view.

A particular mention is worth the family of dataset related to the problem of person detection, tracking, and re-identification from UAV images. The pioneer work is in [171], where the *PRID-2011* dataset is introduced. The height from which images are taken varies between 20 and 60 m and with 1581 identities. *MRP Drone Dataset* [172] introduces a dataset of images specifically designed for the task of people re-identification from UAV. It consists of about 16,000 frames collected in realistic and challenging indoor and outdoor scenarios. Recently, in [173] the *P-DESTRE* dataset have been introduced. It provides the identity annotation of 256 individuals, flying from heights in the range of about 5–7 m, and across multiple days and different appearances.

## 7. Aerial Imaging

**Figure 5:**
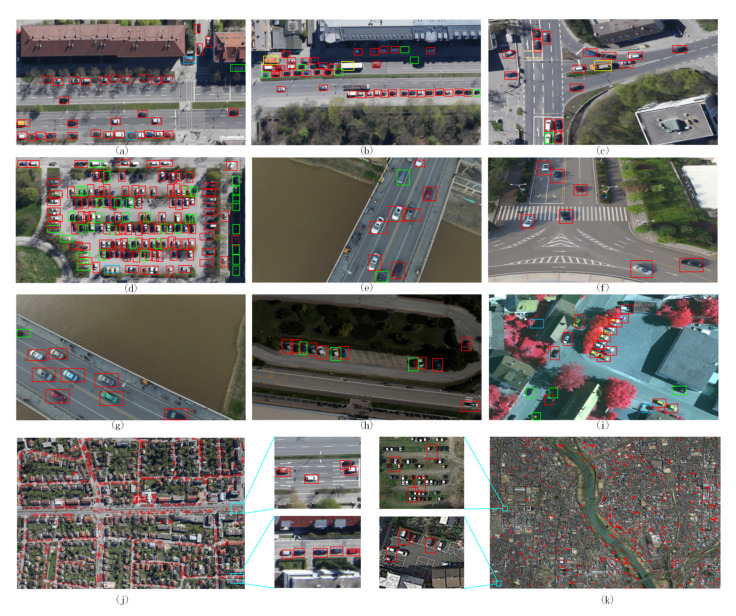
Some outstanding object detection results from the work in [178]. Red boxes denote correct localization of car, yellow boxes denote correct localization of truck, green boxes and blue boxes denote missing detection and incorrect detection, respectively. (**a**–**d**) are results for the DLR 3K test aerial image blocks; (**e**–**h**) are results on unmanned aerial vehicle (UAV) images; (**i**) is results for pansharpened color infrared (CIR) image; (**j**) is results for the original large-scale DLR 3K test image; (**k**) is results for a large satellite image of Tokyo.

A variety of land use tasks, such as urban planning, surveillance, crop monitoring, flood and fire prevention, change detection, traffic monitoring etc., benefits on processing of aerial image processing. The necessity of automatic extraction of valuable information from aerial images encouraged the development and further improvement of various processing methods with a specific purpose. Detecting objects in aerial images is challenged by variance of object colors, aspect ratios, cluttered backgrounds, and in particular, undetermined orientations. In particular objects in multiple orientations have large appearance variation, which challenges existing feature representation and object detection approaches. In addition, the aspect ratios of objects vary with their orientations, which introduces difficulty to object localization. One of the most common tasks in this area concerns detecting the cars in the images. This is particularly challenging due to the relatively small size of the target objects and the complex background in man-made areas. Besides, it requires near-real-time computation in order to provide important information for traffic management and urban planning. In seminal research, image content was described using local and global features. Texture was recognized to have a good discriminative characteristic; thus, it was often used in aerial image classification. Feature extraction methods were efficiently implemented to color, multispectral, and hyperspectral images, and further improvement in classification accuracy was made by post-processing feature data in order to more efficiently model the semantic image content. Despite the fact that feature-based methods had satisfactory performance, they were not exploiting higher order local features and complex spatial dependence between them. Feature describing multi-oriented objects can help traditional approaches, such as in [174]. Some outstanding results in detecting multi-oriented objects results are reported in Figure 5. Implementation of CNNs enabled overcoming of previous drawbacks, with the cost of high time and computational consumption and necessity for sufficient amount of training data. In general, both feature-based and network-based approaches can achieve state-of-the-art performance in the task of aerial image classification under certain conditions. It is worth noting that although in recent years several deep learning based frameworks were proposed for object detection [175], they cannot be exploited as they are conceived and then it is useful also to analyze the ways in which CNNs are adapted for this specific research field. To achieve a useful level of accuracy, additional training data are required in the target domain, but adding this step is quite costly. *Domain adaptation* is a solution used to address this problem but it is still in its infancy in this research field [48]. In addition, self-supervised learning, combined with a more effective representation learning method suitable to remote-sensing problems can be a possible way to explore in order to increase accuracy. Another pathway being explored is the advanced data augmentation, such as the one proposed in [176]. The proposed framework aims at generating annotated objects from remote sensing: it has one generator and two discriminators, which try to synthesize realistic vehicles and learn the background context simultaneously. Finally, a novel detector was proposed in [177] to tackle the challenge of small-scale object detection in aerial images. Based on our observation, it is designed to include an up-scale feature aggregation strategy and a much more efficient cascade up-sampling module for high-resolution features reconstruction.

### 7.1. Vehicle Detection

Vehicle detection in aerial images is a crucial image processing step for many applications like screening of large areas. Most of the existing frameworks of vehicle detection in aerial surveillance are either region-based or sliding window-based. One of the rare pixelwise classification methods for vehicle detection was introduced in [179].

Traditional methods are based on handcrafted features, which cannot reach an optimal balance between the discriminability and the robustness without considering the details of real data. However, some works still deserve to be mentioned for their particular application objective and for their innovative way of dealing with it. As an example the goal of the framework in [180] was to automatically detect vehicles and heavy digging equipment which represent a potentially catastrophic threat to the vast network of oil and gas pipelines in rural areas. The system described is an attempt to allow unmanned airborne vehicles flying at more elevated heights to automatically detect ground vehicles in rural areas. The first stage of the algorithm inspects every image location at several scales and efficiently eliminates the large majority of the background areas. The algorithm begins by quickly detecting features using the Harris corner detector. Next, areas containing a high density of features are detected. The third step clusters heavily overlapping responses. In the final step, color-based properties are used to further refine the results. Another relevant work is the one in [181] where a simple, fast, and high-quality objectness measure by using 8×8 binarized normed gradients features was introduced to handle object scale and aspect ratio by a few atomic (i.e., ADD, BITWISE, etc.) operations. This makes it suitable for many real-time vision applications but other additional cues are required to further reduce the number of object proposals at the early stage.

Recently, vehicle detection methods have been drastically improving due to the use of deep learning. A current prevalent method uses a region-based detector, which searches possible object locations based on image features and classifies them by using a CNN. Several works just apply backbone nets to the extracted regions without adaptation of the models. However, here it is more useful to focus instead on works that introduce models specifically designed to detect vehicles in aerial images.

The extraction of multiscale features at its highest convolutional layer is the key idea in [182], that is one of the milestone work in this research area. Unfortunately, most of the detectors have been developed for datasets that considerably differ from aerial images and this limit their performance in the considered application context. For this reason, in [183], a systematic investigation about the potential of existing detectors has been performed and Fast R-CNN and Faster R-CNN specifically built for aerial images are introduced, achieving top performing results on common detection benchmark datasets. The experimental results on the DLR 3K - Munich Vehicle Aerial Image Dataset (average precision 91.8%) and VEDAI—Vehicle Detection in Aerial Imagery dataset (89.5% on VEDAI 512 and 95.2% on VEDAI 1024) demonstrated indeed the applicability of Fast R-CNN and Faster R-CNN for vehicle detection in aerial images. Intersection over Union (IoU) was used as metric. Although the faster region-based convolutional neural networks (R-CNNs) model has achieved great success in the field of computer vision, several challenges in aerial images limit its applications in vehicle detection. However, a framework based on a single model (even on R-CNN models) do not work well on small targets, do not consider annotation of multiple attributes for targets and available manual annotation of vehicles for training faster R-CNN are not sufficient in number. To overcome these limitations, a more complex framework has been setup in [184]. The framework combines two CNNs: the first one for vehicle-like region proposal (namely Accurate Vehicle Proposal Network, AVPN) based on hyper feature map which combines hierarchical feature maps that are more accurate for small object detection, and a attributes learning network for attribute annotation. Comprehensive evaluations on the public Munich vehicle dataset and the collected vehicle dataset demonstrate the accuracy and effectiveness of the proposed method (recall 77.02% Precision 87.81% F1-score 0.82 computational time per image 3.65 s). Of course the method is slower than those based on a single model and still produces some false, as well as missing detection. Similarly in [178] an improved detection method based on Faster R-CNN is proposed. At first, in order to improve the recall, a Hyper Region Proposal Network (HRCNN) to extract vehicle-like targets with a combination of hierarchical feature maps is used. Then, the classifier after the region proposal network is replaced by a cascade of boosted classifiers to verify the candidate regions, aiming at reducing false detection by negative example mining. Two data sets were used in these experiments. Experiments were performed on DLR 3K—Munich Vehicle Aerial Image Dataset (Recall 78.3%, Precision 89.2% F1-score 0.83 and time per image 3.93 s) and on a collected vehicle data set contains a total of 17 UAV images and 85 very-high-spatial-resolution pansharpened color infrared (CIR) images. Unfortunately building the hyper feature map is not trivial (it should be enhanced with additional significant features) and a possible way to overcome limitations could be of using deconvolution layers to improve the detection performance.

Work in [185] presents a method that can detect the vehicles on a 21-MPixel original frame image without accurate scale information within seconds on a laptop single threaded. It provides also orientation and type (car/truck) information. The approach consists of two step: a fast binary AdaBoost Classifier in a soft-cascade structure and a multiclass classifier on the output of the binary detector, which gives the orientation and type of the vehicles. The method was evaluated on DLR 3K—Munich Vehicle Aerial Image Dataset (Recall 69.3%, Precision 86,8%) and a data set captured from an unmanned aerial vehicle (Recall 79%, Precision 94%). It is a very fast approach but performance are not satisfactory and then it should be improved by using a deep neural network after the binary detector.

The authors in [186] explored orientation-robust features from combined layers of deep CNN. They use CNN trained with ImageNet to extract multiple layers of features. Three deep layers are considered for feature extraction robust to the rotation. At first a graph-cut-based image segmentation approach is used to coarsely localize object candidates, which are then classified with an SVM classifier trained on the orientation invariant features. The proposed approach has been tested on a vehicle dataset and a plane dataset collected from Google Earth aerial images. The vehicle dataset contains 310 images with 2819 vehicle samples. The plane dataset contains 600 images, with 3210 plane samples. Each dataset is split into two subsets: (250 images, 60 images), (500 images, 100 images). One subset is for training, and the other for testing. In vehicle detection, the method achieved a 0.945 precision rate with a 0.8 recall rate. In plane detection, it achieved a 0.972 precision rate with a 0.9 recall rate. Anyway the ability to detect more kinds of aerial objects hasn’t be proved and then it needs further investigation. Summing up, vehicle detection from aerial images is still an ongoing research field and many efforts are still required to get high precision approaches, working in real time on very high resolution images.

### 7.2. Maps Labelling–Semantic Land Classification

The goal is to produce a complete semantic segmentation of an aerial image into classes, such as building, road, tree, grass, and water. It can be an efficient tool for accelerating many applications, like urban development analysis and map updating, also providing great support in crisis and disaster management, and in aiding municipalities in long-term residential area planning. A deeper analysis could also focus on the automatic land-use mapping for change detection, statistics, resources optimization, or economic forecasting scopes. As manual annotation in remotely sensed images is time consuming and unfeasible, researchers focused on automated processing techniques, which can handle various image characteristics and huge amount of data. The availability of accurately labeled data for training tends to be the limiting factor. Hand-labeled data tends to be reasonably accurate, but the cost of hand-labeling and the lack of publicly available hand-labeled datasets strongly restricts the size of the training and test sets for aerial image labeling tasks. Various studies have extracted map parts from high-resolution remotely sensed images using two main techniques, namely data-driven and heuristic methods. Data-driven methods generally use the information of large data to conduct part extraction from satellite images. By contrast, heuristic methods involve texture progressive analysis and mathematical morphology, and often use certain information about targets (road, rivers, wood, crops). Thus, these approaches are useless in handling different types of targets compared with data-driven techniques [187].

Roads are among the most investigated targets to be detected from aerial images. One of the milestone in this specific research area is the work in [188]. The authors, by using synthetic road/non-road labels and a consumer GPU board, were able to efficiently train larger neural networks on much more data than was feasible before. The resulting road detection system works reliably on two large datasets covering a large metropolitan area with both urban and suburban regions. On the one side, the approach looks at a much larger context than was used in previous attempts deriving this way context knowledge that increases discrimination capabilities. On the other side, it relies on the selection of thresholds to make concrete predictions. From that moment the research speeded-up thanks to different deep learning methods.

Among several works in the literature, it is worth mentioning that the one in [189] extended the U-Net [190] model, formerly introduced for fast and precise segmentation of images, by using the deep residual unit as the basis for road detection. The skip connections within the residual units and between the encoding and decoding paths of the network facilitate information propagation both in forward and backward computations allowing the better representation also of context information. This pushed many other pieces of research about the exploitation of residual architectures. For example in [191], a model, namely Refined Deep Residual Convolutional Neural Network (RDRCNN), based on ResNet and U-Net architectures with dilated convolution operators, is used. The method gathered good performance in the task of road extraction from complex backgrounds (city and countryside), but it requires additional processing to more accurately outline boundaries, especially in urban areas.

Another relevant topic is the automatic urban area and building detection. A wide range of publications is available in remote sensing for this topic, often based on shape estimation or contour outlining. There are two different strategies: detecting urban area as a whole or their components (streets, buildings, etc). For detecting urban areas as a traditional and common approach is to build morphological profile to account structural image information and then to apply feature extraction and neural network for classifying the features [192].

Since urban areas show versatile characteristics in remote sensing optical images, multiple features can be utilized to characterize urban areas and this can be achieved by exploiting a multiple conditional random-field ensemble model that incorporate multiple features and learn their contextual information [193].

The method proposed in [194] extracts feature points in the first step by a modified version of Harris corner detector [195], it is automatic and it is able to recognize not just corners but edges as well. After having a local feature point set, a voting matrix strategy is exploited to to get a probability map of the urban area and, finally, an adaptive decision making is performed to find urban areas. Therefore, although it gives an efficient tool for characterizing contour-rich regions, such as urban areas, it does not extract building contours.

A framework performing building detection based on region orientation is the contribution of the work in [196]. It relies on the active contour calculation, which is a computationally intensive part needing implementation tricks and specialized hardware to work in real time.

In [197] a framework that takes raw pixel values in aerial imagery as input and outputs predicted three-channel label images (building–road–background) has been proposed. Using CNNs, both feature extractors and classifiers are automatically constructed. The authors propose a new technique to train a single CNN efficiently for extracting multiple kinds of objects simultaneously.

Finally, in [198,199] a thermal camera is used to detect humans and to calculate Earth’s surface temperature with a high spatiotemporal resolution. These are the only two works dealing with thermal data from aerial images, so this is an open research area that could bring to very precise detection of living beings and to advanced analysis of the ground.

### 7.3. Datasets

Object detection is an important and challenging problem in computer vision. Although the past decade has witnessed major advances in object detection in natural scenes, such successes have been slow to aerial imagery, not only because of the huge variation in the scale, orientation and shape of the object instances on the earth’s surface, but also due to the scarcity of well-annotated datasets of objects in aerial scenes.

To advance object detection research from aerial images different datasets have been recently collected and the most used ones are listed in the followings.

*DOTA—large-scale Dataset for Object deTection in Aerial images* [200], has been introduced in 2018. It collects 2806 aerial images from different sensors and platforms. Each image is of the size about 4000-by-4000 pixels and contains objects exhibiting a wide variety of scales, orientations, and shapes. These DOTA images are then annotated by experts in aerial image interpretation using 15 common object categories (plane, ship, storage tank, baseball diamond, tennis court, swimming pool, ground track field, harbor, bridge, large vehicle, small vehicle, helicopter, roundabout, soccer ball field and basketball court). The fully annotated DOTA images contains 188,282 instances, each of which is labeled by an arbitrary (8 d.o.f.) quadrilatera.

*DLR 3K—Munich Vehicle Aerial Image Dataset* (https://pba-freesoftware.eoc.dlr.de/MunichDatasetVehicleDetection-2015-old.zip) [185] Images were captured by the DLR 3K camera system over the area of Munich, Germany. The 3K camera consists of three Canon 1Ds Mark II cameras with a 36×24 mm CMOS chip at 16.7 Megapixels and a 50 mm focal length. Spatial resolution was 5616×3744 pixels and images were stored in JPEG format. The maximum frame rate is 3 fps. The cameras are arranged to provide one nadir view and two oblique views. The flight height was 1000 m above ground, the ground sampling distance is approximately 15 cm. Munich Vehicle dataset is annotated with rotated bounding boxes for seven types of vehicles: bus, car, car with trailer, truck, truck with trailer, van with trailer, long truck.

*VEDAI—Vehicle Detection in Aerial Imagery* [201]: has been built by retaining the images acquired by the satellite Utah AGRC and in particular the photography set set HRO 2012 6 in., The images were taken during spring 2012. A total of 1210 images (RGB or infrared) have been manually selected and made available in two resolutions: 1024×1024 pixels (namely VEDAI 1024) and 512×512 pixels (namely VEDAI 512). All images have been taken from the same distance to the ground. The dataset has many different type of backgrounds (e.g., fields, grass, mountains, urban area, etc.). The proposed dataset contains nine different classes of vehicles, namely the “plane”, “boat”, “camping car”, “car”, “pick-up”, “tractor”, “truck”, “van”, and the “other” category. There is an average of 5.5 vehicles per image, and they occupy about 0.7% of the total pixels of the images.

*DIOR—DetectIon in Optical Remote sensing images* [175] consists of 23,463 images covered by 20 object categories and each category contains about 1200 images. The objects have a large range of size variations, not only in terms of spatial resolutions, but also in the aspect of inter- and intra-class size variability across objects. The dataset holds large variations because the images are obtained with different imaging conditions, weathers, seasons, and image quality.

*COWC—Cars Overhead with Context* dataset [202] is a large, high quality set of annotated cars from overhead imagery. The data consists of 33,000 unique cars from six different image locales.

*URBAN—Massachusetts roads and buildings dataset* [188] consists of urban aerial imagery at a resolution of 1.2 m per pixel. The dataset covers a large metropolitan area with both urban and suburban regions. It consists of a training set that covers roughly 500 km${}^{2}$, a separate test set of 50 km${}^{2}$, and a separate small validation set that was used for model selection. There is also another testing subset covering 28 km${}^{2}$ of aerial imagery of a different city. The buildings dataset consists of 151 aerial images of the Boston area whereas the roads dataset consists of 1171 aerial images of the state of Massachusetts.

## 8. Discussion

Although each category has shown proper peculiarities, it is useful to make some considerations that are shared between the different taxonomies evaluated in this manuscript.

Differently from classical computer vision and machine learning methodologies that for reaching satisfying results often required complex handcrafted engineered solutions, the advent of deep learning has shown that impressive outcomes for real-world applications could be obtained with even simple end-to-end direct pipelines. Such progress has been, most probably, the consequence of two principal factors: on the one hand, the publications of datasets for object detection relevant to UAV research, which made available common benchmark platforms and possible to train deep networks with supervision using huge collections of labeled data; on the other hand, the evolution of general-purpose 2D object detection network architectures that have incorporated ideas from classic computer vision interpreting such instruments in light of deep learning computation.

Notwithstanding these generic considerations, it is important also to derive specific consequences that are related to the specifically defined interval of height. About eye level view, it has been already observed how flying at men heights means to be in the same conditions, in terms of viewpoint and appearance, of many classic computer vision problems. This has two important implications. The first one is that the results of many well-investigated solutions not specifically designed for the case of UAV, e.g., pedestrian detection [203], could be applied. Nevertheless, this domain presents specific challenges: the first problem regards how efficiently shift the computation on-board, i.e., with limited computational capabilities, and this leads to the preference for mobile architecture or adapting pre-existing backbones. An alternative is to distribute computation, and in this case, the bottleneck becomes the search for efficient data transmission schemes. Other challenges are more related to the image appearance, i.e., how to continue detecting the object in the case of fast-moving cameras, different object scales, and/or the detection in multiple views. The second implication is that, similarly, datasets designed for ground computer vision and robotics are usually re-used in this domain. If this has allowed to rapidly shift deep learning-based object detection on-board of the UAVs, from the other side, it could represent a bottleneck for the portability of such systems in realistic scenarios.

As a very qualitative tendency, in the case of UAVs with very limited computational resources and/or in the cases of vision-based control, the methods still can rely on *classic* object descriptor. Deep learning is privileged in more expensive solutions, or in the cases where the images are processed offline and/or off-board. The computational burden of these models is still high when applied to the UAV’s obstacle avoidance. Also, it is proved that part of the computation is not required since the blocks with a smaller number of layers could yield reliable results for the UAV’s obstacle avoidance [101].

Finally, flying at such heights necessarily means to interact with people. In this regard, HDI represents a very mature sector, with many works focusing on different aspects. In this multidisciplinary field, active research aims at integrating functionalities from both the engineering and the social interaction parts, as well as the creation of companion UAVs [204].

In the case of low and medium flying heights, the direction that the research community has more recently undertaken preeminently regards the use of deep learning to overcome the challenges posed by object detection from the point of view of a UAV mounted camera. In this regard, numerous datasets and international challenges are arising. Thus, it is possible to foresee that low and medium heights will have the most benefit from deep learning advancements, with a considerable boost that can be expected in the next few years. Anyway, at the present, the adoption of more classical and engineered computer vision pipelines is still not rare, especially considering the lack of adequate computing resources, such as embedded GPU, or caused by the absence of labeled data for a specific task/environment.

Aerial image analysis has been boosted too by deep learning. Anyway, new specific challenges need to be addressed: a trade-off between network complexity and processing time has to be defined considering the aerial images have high spatial resolution and, at the same time, they can frame a huge informative content to be examined. This is mainly true in the case of vehicle detection tasks, where quick outcomes can also be required to reach the application goal. Concerning maps labeling, the main issues are related to the ability of the methods to extract contextual information that is the key to rightly classify the objects (bridges, buildings, roads) that have a very similar appearance. Finally, for semantic land classification, the exploitation of new sensors (e.g., thermal or multi-spectral cameras) has paved the pathway towards new research lines.

A summary of the principal works cited in this document is reported in Table 1. For each entry, we sum up the taxonomy, the network used to perform object detection, and the obtained performance on the specific dataset. Performance is reported in terms of mAP if not differently specified.

The aforementioned observations lead us to conclude that the fundamental means to evaluate and improve the performance of object detectors is related to the availability of public datasets containing labeled images. Data is one of the fundamental components for performing deep learning architectures, and the UAVs do not represent an exception. A summary of the dataset introduced in this review is provided in Table 2. It is possible to observe that for both the different analyzed ranges and application domains, there exist specific datasets that can let data comparison schemes. Anyway, in particular, at eye level heights, it is possible to observe that there are cases where the UAV motion is only simulated since surveillance cameras are often used. Moreover, many works validate their hypotheses on classic object recognition datasets not specifically designed from the UAV; if this is partially motivated by the viewpoint sharing at such heights, it is also true that this can affect their effective use in realistic scenarios, since motion blur, changes in scale and viewpoints that are typical from the UAV framing, are only partially considered with this approach. On the contrary, the field of aerial imaging presents more well-established datasets that are often used in terms of comparison of each proposed processing pipeline.

The analysis as a whole of the considered topic made clear that, in the following years, it would be desirable to have new challenging datasets which take into account multiple heights at the same time, as well as object observed from different scales and viewpoints. This will contribute to consolidating the research findings and allowing trusting computational outcomes even in critical application fields.

Finally, in this work, some reference to works adding 3D information and integrating different sensors has been provided. An extensive analysis of UAV object detection basing on these sources of data is out of the scope of this work and it would require a separate study. Anyway, through the proposed review, it was observed how sensors such as thermal imaging and 3D cameras are mature enough to improve the detection performance. Instead, event-based cameras are still at an early stage, providing object detection based on optical flow and motion compensation, but representing at the same time a very promising research direction. The other promising domain is sensor fusion, where both exteroceptive and proprioceptive sensors can enhance performance. Learning from multimodal sensors is a unique possibility to deeply capture correspondences between modalities and gaining an in-depth understanding of the scene. In the next few years, it is possible to expect the arising of deep neural networks that take multimodal input data and specifically designed for the UAV.

## 9. Conclusions

UAVs achieved an unprecedented level of growth in many civil and military application domains, and computer vision has undoubtedly a key role in providing the necessary information concerning what is sensed. In this review, recent advancements in 2D object detection for the case of UAVs were reported on, focusing on the differences, strategies, and trade-offs between the generic problem of object detection, and the adaptation of such solutions for operations of the UAV. In particular, a new taxonomy that considers different heights intervals and driven by the methodological approaches introduced by the works in the state of the art instead of hardware, physical, and/or technological constraints were proposed. In the manuscript, it emerged that the advent of deep learning has shown that impressive outcomes for real-world applications which could be obtained with even simple end-to-end direct pipelines. This drove us to conclude that a fundamental means to evaluate and improve the performance of object detectors is the availability of public datasets containing labeled images. An adequate level of labeled data is already available only for aerial images whereas, for contexts using images taken at lower heights, additional efforts for data collection are still required. A proposed pathway to improve this research area concerns non-heterogeneous data coming from different sensing devices since learning from multimodal sensors gives a great opportunity to deeply capture correspondences between modalities and gaining an in-depth understanding of the scene.

## Figures and Tables

**Table 1:** Summary of selected object detection methodologies at different flight heights. *RP* stands for *Relaxed Precision* as introduced in [188], *cmpp* stands for *cm per pixel* and *mpp* stands for *meters per pixel*.

Taxonomy	Method	Object Detection Network	Metric (mAP)	Objects Size	Dataset
Eye Level	[89] (2017)	handcrafted features	97.4%	8500–200,000 pixels	own data
	[92] (2017)	handcrafted features	76.97% Sensitivity	-	CICTE-PeopleDetection
Eye Level/Low-Medium	[124] (2019)	Tiny Face	96.5% Precision	5–25 pixels inter eye	DroneSURF *active*
	[142] (2019)	Tiny YOLOV3	67%	-	Swimmers
	[142] (2019)	SSD MobileNetV2	21%		
Low-Medium	[165] (2019)	CornerNet	17.41%	3–355,432 pixels	Visdrone *test*
	[165] (2019)	Light-Head R-CNN	16.53%		
	[165] (2019)	FPN	16.51%		
	[167] (2018)	R-FCN	34.35%	29–131,803 pixels	UAVDT
	[167] (2018)	SSD	33.62%		
	[167] (2018)	Faster R-CNN	22.32%		
	[169] (2020)	Tiny YOLOV3	30.22%	9–2,046,720 pixels	AU-AIR
	[169] (2020)	SSDLite MobileNetV2	19.50%		
Aerial	[183] (2017)	Fast R-CNN	95.2%	12.5 cmpp	VEDAI 1024
	[183] (2017)	Fast R-CNN	91.4%	15 cmpp	DLR 3K
	[184] (2017)	AVPN + R-CNN	87.81%		
	[178] (2017)	Hyper R-CNN	89.2%		
	[186] (2015)	AlexNet + SVM	94.5%	15 cmpp	Google Earth aerial images
	[189] (2018)	deep residual U-Net	91.87% RP (roads)	1.2 mpp	URBAN
	[191] (2019)	RDRCNN + post proc.	99.9% (roads)		
	[197] (2016)	CNN	92.30% (buildings)		

**Table 2:** A summary of relevant datasets for 2D object detection from UAV and aerial images. *n.d.* stands for *no data*.

Dataset	Taxonomy	Task	Heights Range	# Images	Resolution	Notes
CICTE-PeopleDetection [92]	Eye Level	Pedestrian Detection	2.3–5 m	43K+	50×100	emulates UAV with videos from 100 surveillance cameras
DroneSURF [124]	Eye Level/Low-Medium	Face Detection and Recognition	n.d.	411K+	1280×720	DJI Phantom 4; 58 subjects with more than 786K face annotations
Swimmers [142]	Eye Level/Low-Medium	People (Swimmers) Detection	n.d.	9000	1920×1080	mix of labeled internet pictures with images from a UAV flying over the sea shore
UAV123 [163]	Low-Medium	Object Detection and Tracking	5–25 m	110K+	1280×720	additional scenes taken from a simulation from Unreal4 Game Engine
Visdrone [164]	Low-Medium	Object Detection	various heights	8599	1920×1080	wide-range of flight height and viewpoint; huge number of labeled bounding boxes (540K)
UAVDT [167]	Low-Medium	Object Detection	10–70+ m	80K	1080×540	labeled weather conditions; wide-range of flight height; 3 camera views
P-DESTRE [173]	Low-Medium	Pedestrian Detection, Tracking and Re-Identification	5.5–6.7 m	n.d.	3840×2160	DJI Phantom 4; 269 identities with 16 biometrics labels
Stanford Campus [168]	Low-Medium	Object Tracking and Trajectory Forecasting	~80 m	920K+	1400×1904	3DR SOLO; annotated interactions between targets and space
AU-AIR [169]	Low-Medium	Object Detection	10–30 m	32K+	1920×1080	Parrot Bepop2; multi-modal, contains GPS, Altitude, Velocity, IMU, and Time
DOTA [200]	Aerial	Object Detection	n.d.	2806	800×800–4000×4000	collected from Google Earth and other aerial photography platforms; 15 object categories, from vehicles to field types and buildings
DLR 3K [185]	Aerial	Vehicle Detection	1000+ m	20	5616×3744	DLR 3K camera system: three Canon 1Ds Mark II on-board of Dornier DO 228 or a Cessna 208B Grand Caravan; only 3418 cars and 54 trucks labels
VEDAI [201]	Aerial	Vehicle Detection	n.d.	1210	512×512, 1024×1024	collected from Utah AGRC (http://gis.utah.gov/) aerial photography set; RGB plus near infrared channel; 9 vehicle classes
DIOR [175]	Aerial	Object Detection	n.d.	23K+	800×800	collected from Google Earth; labeled with 20 object categories
COWC [202]	Aerial	Car Detection	n.d.	388K+	256×256	six aerial photography sets from different regions in the world (Germany, New Zealand, USA, Canada); more than 30k unique cars annotated
URBAN [188]	Aerial	Buildings and Roads Detection	n.d.	1322	1500×1500	mostly urban and suburban areas and buildings of all sizes, including individual houses and garages

## Abbreviations

The following abbreviations and acronyms are used in this manuscript:

CNN	Convolutional Neural Network
FAST	Features from Accelerated Segment Test
FPN	Feature Pyramid Network
GCS	Ground Control Station
GPS	Global Positioning System
GPU	Graphics Processing Unit
IMU	Inertial Measurement Unit
INS	Inertial Navigation System
LiDAR	Light Detection and Ranging
LSH	Locality-Sensitive Hashing
mAP	mean Average Precision
ReLU	Rectified Linear Unit
RP	Relaxed Precision
RPA	Remotely Piloted Aircraft
RPAS	Remotely Piloted Aerial System
UAV	Unmanned Aerial Vehicle
UAS	Unmanned Aerial System

## References

[1] Walia K. (2019). VTOL UAV Market 2025 Research Report—Industry Size & Share.

[2] PricewaterhouseCoopers (PwC) (2018). Skies without Limits–Drones-Taking the UK’s Economy to New Heights.

[3] Undertaking S.J. (2016). European Drones Outlook Study—Unlocking the Value for Europe.

[4] Valavanis K.P. (2008). Advances in Unmanned Aerial Vehicles: State of the Art and the Road to Autonomy.

[5] Lu Y., Xue Z., Xia G.S., Zhang L. (2018). A survey on vision-based UAV navigation. Geo-Spat. Inf. Sci..

[6] Kanellakis C., Nikolakopoulos G. (2017). Survey on computer vision for UAVs: Current developments and trends. J. Intell. Robot. Syst..

[7] Roberts L.G. (1963). Machine perception of three-dimensional soups. Mass. Inst. Technol..

[8] Papert S.A. (1966). The Summer Vision Project.

[9] Al-Kaff A., Martin D., Garcia F., de la Escalera A., Armingol J.M. (2018). Survey of computer vision algorithms and applications for unmanned aerial vehicles. Expert Syst. Appl..

[10] Zhao J., Xiao G., Zhang X., Bavirisetti D.P. A Survey on Object Tracking in Aerial Surveillance. Proceedings of the International Conference on Aerospace System Science and Engineering.

[11] Xu Y., Pan L., Du C., Li J., Jing N., Wu J. Vision-based uavs aerial image localization: A survey. Proceedings of the 2nd ACM SIGSPATIAL International Workshop on AI for Geographic Knowledge Discovery.

[12] Carrio A., Sampedro C., Rodriguez-Ramos A., Campoy P. (2017). A review of deep learning methods and applications for unmanned aerial vehicles. J. Sensors.

[13] Nikolakopoulos K.G., Soura K., Koukouvelas I.K., Argyropoulos N.G. (2017). UAV vs classical aerial photogrammetry for archaeological studies. J. Archaeol. Sci. Rep..

[14] Hoskere V., Park J.W., Yoon H., Spencer Jr B.F. (2019). Vision-based modal survey of civil infrastructure using unmanned aerial vehicles. J. Struct. Eng..

[15] Karantanellis E., Marinos V., Vassilakis E., Christaras B. (2020). Object-Based Analysis Using Unmanned Aerial Vehicles (UAVs) for Site-Specific Landslide Assessment. Remote Sens..

[16] Tsokos A., Kotsi E., Petrakis S., Vassilakis E. (2018). Combining series of multi-source high spatial resolution remote sensing datasets for the detection of shoreline displacement rates and the effectiveness of coastal zone protection measures. J. Coast. Conserv..

[17] Valavanis K.P., Vachtsevanos G.J. (2015). Handbook of Unmanned Aerial Vehicles.

[18] Commission Directive (EU) 2019/514 of 14 March 2019 Amending Directive 2009/43/EC of the European Parliament and of the Council as Regards the List of Defence-Related Products (Text with EEA Relevance). https://eur-lex.europa.eu/legal-content/EN/TXT/?uri=uriserv:OJ.L_.2019.089.01.0001.01.ENG.

[19] Siciliano B., Khatib O. (2016). Springer Handbook of Robotics.

[20] Commission Implementing Regulation (EU) 2019/947 of 24 May 2019 on the Rules and Procedures for the Operation of Unmanned Aircraft (Text with EEA Relevance). https://eur-lex.europa.eu/legal-content/EN/TXT/?uri=CELEX:02019R0947-20200606.

[21] Cuerno-Rejado C., García-Hernández L., Sánchez-Carmona A., Carrio A., Sánchez-López J.L., Campoy P. (2016). Historical Evolution of the Unmanned Aerial Vehicles to the Present. DYNA.

[22] Dalamagkidis K., Valavanis K.P., Vachtsevanos G.J. (2015). Classification of UAVs. Handbook of Unmanned Aerial Vehicles.

[23] Baca T., Stepan P., Saska M. Autonomous landing on a moving car with unmanned aerial vehicle. Proceedings of the 2017 European Conference on Mobile Robots (ECMR).

[24] Mitrokhin A., Fermüller C., Parameshwara C., Aloimonos Y. Event-based moving object detection and tracking. Proceedings of the 2018 IEEE/RSJ International Conference on Intelligent Robots and Systems (IROS).

[25] Afshar S., Ralph N., Xu Y., Tapson J., Schaik A.v., Cohen G. (2020). Event-based feature extraction using adaptive selection thresholds. Sensors.

[26] Gade R., Moeslund T.B. (2014). Thermal cameras and applications: A survey. Mach. Vis. Appl..

[27] Portmann J., Lynen S., Chli M., Siegwart R. People detection and tracking from aerial thermal views. Proceedings of the 2014 IEEE international conference on robotics and automation (ICRA).

[28] Ramon Soria P., Arrue B.C., Ollero A. (2017). Detection, location and grasping objects using a stereo sensor on uav in outdoor environments. Sensors.

[29] Mashood A., Noura H., Jawhar I., Mohamed N. A gesture based kinect for quadrotor control. Proceedings of the 2015 International Conference on Information and Communication Technology Research (ICTRC).

[30] Yu Y., Wang X., Zhong Z., Zhang Y. ROS-based UAV control using hand gesture recognition. Proceedings of the 2017 29th Chinese Control And Decision Conference (CCDC).

[31] Shao L., Han J., Kohli P., Zhang Z. (2014). Computer Vision and Machine Learning with RGB-D Sensors.

[32] Hall D.L., McMullen S.A. (2004). Mathematical Techniques in Multisensor Data Fusion.

[33] Sanchez-Lopez J.L., Arellano-Quintana V., Tognon M., Campoy P., Franchi A. (2017). Visual marker based multi-sensor fusion state estimation. IFAC-PapersOnLine.

[34] Sanchez-Lopez J.L., Molina M., Bavle H., Sampedro C., Fernández R.A.S., Campoy P. (2017). A multi-layered component-based approach for the development of aerial robotic systems: The aerostack framework. J. Intell. Robot. Syst..

[35] Endsley M.R. (1995). Toward a theory of situation awareness in dynamic systems. Hum. Factors.

[36] Siegwart R., Nourbakhsh I.R., Scaramuzza D. (2011). Introduction to Autonomous Mobile Robots.

[37] Rosinol A., Gupta A., Abate M., Shi J., Carlone L. (2020). 3D Dynamic Scene Graphs: Actionable Spatial Perception with Places, Objects, and Humans. arXiv.

[38] Bavle H., De La Puente P., How J.P., Campoy P. (2020). VPS-SLAM: Visual Planar Semantic SLAM for Aerial Robotic Systems. IEEE Access.

[39] Zhang L., Wei L., Shen P., Wei W., Zhu G., Song J. (2018). Semantic SLAM based on object detection and improved octomap. IEEE Access.

[40] Manzoor S., Joo S.H., Rocha Y.G., Lee H.U., Kuc T.Y. A Novel Semantic SLAM Framework for Humanlike High-Level Interaction and Planning in Global Environment. Proceedings of the 1st International Workshop on the Semantic Descriptor, Semantic Modeling and Mapping for Humanlike Perception and Navigation of Mobile Robots toward Large Scale Long-Term Autonomy (SDMM1).

[41] Sanchez-Lopez J.L., Sampedro C., Cazzato D., Voos H. Deep learning based semantic situation awareness system for multirotor aerial robots using LIDAR. Proceedings of the 2019 International Conference on Unmanned Aircraft Systems (ICUAS).

[42] Sanchez-Lopez J.L., Castillo-Lopez M., Voos H. Semantic situation awareness of ellipse shapes via deep learning for multirotor aerial robots with a 2D LIDAR. Proceedings of the 2020 International Conference on Unmanned Aircraft Systems (ICUAS).

[43] Lin Y., Saripalli S. (2017). Sampling-based path planning for UAV collision avoidance. IEEE Trans. Intell. Transp. Syst..

[44] Sanchez-Lopez J.L., Wang M., Olivares-Mendez M.A., Molina M., Voos H. (2019). A real-time 3d path planning solution for collision-free navigation of multirotor aerial robots in dynamic environments. J. Intell. Robot. Syst..

[45] Castillo-Lopez M., Ludivig P., Sajadi-Alamdari S.A., Sanchez-Lopez J.L., Olivares-Mendez M.A., Voos H. (2020). A Real-Time Approach for Chance-Constrained Motion Planning With Dynamic Obstacles. IEEE Robot. Autom. Lett..

[46] Pestana J., Sanchez-Lopez J.L., Saripalli S., Campoy P. Computer vision based general object following for gps-denied multirotor unmanned vehicles. Proceedings of the 2014 American Control Conference.

[47] Pestana J., Sanchez-Lopez J.L., Campoy P., Saripalli S. Vision based gps-denied object tracking and following for unmanned aerial vehicles. Proceedings of the 2013 IEEE International Symposium on Safety, Security, and Rescue Robotics (SSRR).

[48] Koga Y., Miyazaki H., Shibasaki R. (2020). A Method for Vehicle Detection in High-Resolution Satellite Images that Uses a Region-Based Object Detector and Unsupervised Domain Adaptation. Remote Sens..

[49] Redding J.D., McLain T.W., Beard R.W., Taylor C.N. Vision-based target localization from a fixed-wing miniature air vehicle. Proceedings of the 2006 American Control Conference.

[50] Semsch E., Jakob M., Pavlicek D., Pechoucek M. Autonomous UAV surveillance in complex urban environments. Proceedings of the 2009 IEEE/WIC/ACM International Joint Conference on Web Intelligence and Intelligent Agent Technology.

[51] Nikolic J., Burri M., Rehder J., Leutenegger S., Huerzeler C., Siegwart R. A UAV system for inspection of industrial facilities. Proceedings of the 2013 IEEE Aerospace Conference.

[52] Sankey T., Donager J., McVay J., Sankey J.B. (2017). UAV lidar and hyperspectral fusion for forest monitoring in the southwestern USA. Remote Sens. Environ..

[53] Cazzato D., Olivares-Mendez M.A., Sanchez-Lopez J.L., Voos H. Vision-Based Aircraft Pose Estimation for UAVs Autonomous Inspection without Fiducial Markers. Proceedings of the IECON 2019—45th Annual Conference of the IEEE Industrial Electronics Society.

[54] Andreopoulos A., Tsotsos J.K. (2013). 50 years of object recognition: Directions forward. Comput. Vis. Image Underst..

[55] Liu L., Ouyang W., Wang X., Fieguth P., Chen J., Liu X., Pietikäinen M. (2020). Deep learning for generic object detection: A survey. Int. J. Comput. Vis..

[56] Ponce J., Hebert M., Schmid C., Zisserman A. (2007). Toward Category-Level Object Recognition.

[57] Russakovsky O., Deng J., Su H., Krause J., Satheesh S., Ma S., Huang Z., Karpathy A., Khosla A., Bernstein M. (2015). Imagenet large scale visual recognition challenge. Int. J. Comput. Vis..

[58] Wang R., Xu J., Han T.X. (2019). Object instance detection with pruned Alexnet and extended training data. Signal Process. Image Commun..

[59] Viola P., Jones M. Rapid object detection using a boosted cascade of simple features. Proceedings of the 2001 IEEE Computer Society Conference on Computer Vision and Pattern Recognition (CVPR).

[60] Dalal N., Triggs B. Histograms of oriented gradients for human detection. Proceedings of the 2005 IEEE Computer Society Conference on Computer Vision and Pattern Recognition (CVPR’05).

[61] Rublee E., Rabaud V., Konolige K., Bradski G. ORB: An efficient alternative to SIFT or SURF. Proceedings of the 2011 International Conference on Computer Vision.

[62] Zou Z., Shi Z., Guo Y., Ye J. (2019). Object detection in 20 years: A survey. arXiv.

[63] Krizhevsky A., Sutskever I., Hinton G.E. (2012). Imagenet classification with deep convolutional neural networks. Advances in Neural Information Processing Systems.

[64] Deng J., Dong W., Socher R., Li L.J., Li K., Fei-Fei L. Imagenet: A large-scale hierarchical image database. Proceedings of the 2009 IEEE Conference on Computer Vision and Pattern Recognition.

[65] Girshick R., Donahue J., Darrell T., Malik J. Rich feature hierarchies for accurate object detection and semantic segmentation. Proceedings of the IEEE Conference on Computer Vision and Pattern Recognition.

[66] Ren S., He K., Girshick R., Sun J. (2015). Faster r-cnn: Towards real-time object detection with region proposal networks. Advances in Neural Information Processing Systems.

[67] He K., Gkioxari G., Dollár P., Girshick R. Mask r-cnn. Proceedings of the IEEE International Conference on Computer Vision.

[68] Redmon J., Divvala S., Girshick R., Farhadi A. You only look once: Unified, real-time object detection. Proceedings of the IEEE Conference on Computer Vision and Pattern Recognition.

[69] Redmon J., Farhadi A. (2018). Yolov3: An incremental improvement. arXiv.

[70] Lin T.Y., Dollár P., Girshick R., He K., Hariharan B., Belongie S. Feature pyramid networks for object detection. Proceedings of the IEEE Conference on Computer Vision and Pattern Recognition.

[71] Iandola F.N., Han S., Moskewicz M.W., Ashraf K., Dally W.J., Keutzer K. (2016). SqueezeNet: AlexNet-level accuracy with 50× fewer parameters and <0.5 MB model size. arXiv.

[72] Gholami A., Kwon K., Wu B., Tai Z., Yue X., Jin P., Zhao S., Keutzer K. Squeezenext: Hardware-aware neural network design. Proceedings of the IEEE Conference on Computer Vision and Pattern Recognition Workshops.

[73] Zhang X., Zhou X., Lin M., Sun J. Shufflenet: An extremely efficient convolutional neural network for mobile devices. Proceedings of the IEEE Conference on Computer Vision and Pattern Recognition.

[74] Ma N., Zhang X., Zheng H.T., Sun J. Shufflenet v2: Practical guidelines for efficient cnn architecture design. Proceedings of the European Conference on Computer Vision (ECCV).

[75] Howard A.G., Zhu M., Chen B., Kalenichenko D., Wang W., Weyand T., Andreetto M., Adam H. (2017). Mobilenets: Efficient convolutional neural networks for mobile vision applications. arXiv.

[76] Sifre L. (2014). Rigid-Motion Scattering for Image Classification. Ph.D. Thesis.

[77] Chollet F. Xception: Deep learning with depthwise separable convolutions. Proceedings of the IEEE Conference on Computer Vision and Pattern Recognition.

[78] Sandler M., Howard A., Zhu M., Zhmoginov A., Chen L.C. Mobilenetv2: Inverted residuals and linear bottlenecks. Proceedings of the IEEE Conference on Computer Vision and Pattern Recognition.

[79] Tan M., Chen B., Pang R., Vasudevan V., Sandler M., Howard A., Le Q.V. MnasNet: Platform-Aware Neural Architecture Search for Mobile. Proceedings of the IEEE Conference on Computer Vision and Pattern Recognition (CVPR).

[80] Wu B., Dai X., Zhang P., Wang Y., Sun F., Wu Y., Tian Y., Vajda P., Jia Y., Keutzer K. Fbnet: Hardware-aware efficient convnet design via differentiable neural architecture search. Proceedings of the IEEE Conference on Computer Vision and Pattern Recognition.

[81] Dai X., Zhang P., Wu B., Yin H., Sun F., Wang Y., Dukhan M., Hu Y., Wu Y., Jia Y. Chamnet: Towards efficient network design through platform-aware model adaptation. Proceedings of the IEEE Conference on Computer Vision and Pattern Recognition.

[82] Howard A., Sandler M., Chu G., Chen L.C., Chen B., Tan M., Wang W., Zhu Y., Pang R., Vasudevan V. Searching for mobilenetv3. Proceedings of the IEEE International Conference on Computer Vision.

[83] Elsken T., Metzen J.H., Hutter F. (2018). Neural architecture search: A survey. arXiv.

[84] Wistuba M., Rawat A., Pedapati T. (2019). A survey on neural architecture search. arXiv.

[85] Tan M., Le Q.V. (2019). Efficientnet: Rethinking model scaling for convolutional neural networks. arXiv.

[86] Cheng Y., Wang D., Zhou P., Zhang T. (2017). A survey of model compression and acceleration for deep neural networks. arXiv.

[87] Choudhary T., Mishra V., Goswami A., Sarangapani J. (2020). A comprehensive survey on model compression and acceleration. Artif. Intel. Rev..

[88] Chen G., Choi W., Yu X., Han T., Chandraker M. (2017). Learning efficient object detection models with knowledge distillation. Advances in Neural Information Processing Systems.

[89] Al-Kaff A., García F., Martín D., De La Escalera A., Armingol J.M. (2017). Obstacle detection and avoidance system based on monocular camera and size expansion algorithm for UAVs. Sensors.

[90] Lv Q., Josephson W., Wang Z., Charikar M., Li K. Multi-probe LSH: Efficient indexing for high-dimensional similarity search. Proceedings of the 33rd International Conference on Very Large Data Bases.

[91] Hesch J.A., Roumeliotis S.I. A direct least-squares (DLS) method for PnP. Proceedings of the 2011 IEEE International Conference on Computer Vision (ICCV).

[92] Aguilar W.G., Luna M.A., Moya J.F., Abad V., Parra H., Ruiz H. Pedestrian detection for UAVs using cascade classifiers with meanshift. Proceedings of the 2017 IEEE 11th International Conference on Semantic Computing (ICSC).

[93] De Smedt F., Hulens D., Goedemé T. On-board real-time tracking of pedestrians on a UAV. Proceedings of the IEEE Conference on Computer Vision and Pattern Recognition Workshops.

[94] Peng X.Z., Lin H.Y., Dai J.M. Path planning and obstacle avoidance for vision guided quadrotor UAV navigation. Proceedings of the 2016 12th IEEE International Conference on Control and Automation (ICCA).

[95] McGuire K., De Croon G., De Wagter C., Tuyls K., Kappen H. (2017). Efficient optical flow and stereo vision for velocity estimation and obstacle avoidance on an autonomous pocket drone. IEEE Robot. Autom. Lett..

[96] Lee J., Wang J., Crandall D., Šabanović S., Fox G. Real-time, cloud-based object detection for unmanned aerial vehicles. Proceedings of the 2017 First IEEE International Conference on Robotic Computing (IRC).

[97] Smolyanskiy N., Kamenev A., Smith J., Birchfield S. Toward low-flying autonomous MAV trail navigation using deep neural networks for environmental awareness. Proceedings of the 2017 IEEE/RSJ International Conference on Intelligent Robots and Systems (IROS).

[98] He K., Zhang X., Ren S., Sun J. Deep residual learning for image recognition. Proceedings of the IEEE Conference on Computer Vision and Pattern Recognition.

[99] Schmidhuber J., Huber R. (1991). Learning to generate artificial fovea trajectories for target detection. Int. J. Neural Syst..

[100] Loquercio A., Maqueda A.I., Del-Blanco C.R., Scaramuzza D. (2018). Dronet: Learning to fly by driving. IEEE Robot. Autom. Lett..

[101] Dai X., Mao Y., Huang T., Qin N., Huang D., Li Y. (2020). Automatic Obstacle Avoidance of Quadrotor UAV via CNN-based Learning. Neurocomputing.

[102] Wojciechowska A., Frey J., Sass S., Shafir R., Cauchard J.R. Collocated human-drone interaction: Methodology and approach strategy. Proceedings of the 2019 14th ACM/IEEE International Conference on Human-Robot Interaction (HRI).

[103] Monajjemi M., Bruce J., Sadat S.A., Wawerla J., Vaughan R. UAV, do you see me? Establishing mutual attention between an uninstrumented human and an outdoor UAV in flight. Proceedings of the 2015 IEEE/RSJ International Conference on Intelligent Robots and Systems (IROS).

[104] Karjalainen K.D., Romell A.E.S., Ratsamee P., Yantac A.E., Fjeld M., Obaid M. Social drone companion for the home environment: A user-centric exploration. Proceedings of the 5th International Conference on Human Agent Interaction.

[105] Arroyo D., Lucho C., Roncal S.J., Cuellar F. Daedalus: A sUAV for human-robot interaction. Proceedings of the 2014 ACM/IEEE International Conference on Human-Robot Interaction.

[106] Cauchard J.R., Zhai K.Y., Spadafora M., Landay J.A. Emotion encoding in human-drone interaction. Proceedings of the 2016 11th ACM/IEEE International Conference on Human-Robot Interaction (HRI).

[107] Leo M., Carcagnì P., Mazzeo P.L., Spagnolo P., Cazzato D., Distante C. (2020). Analysis of Facial Information for Healthcare Applications: A Survey on Computer Vision-Based Approaches. Information.

[108] Perera A.G., Wei Law Y., Chahl J. UAV-GESTURE: A dataset for UAV control and gesture recognition. Proceedings of the European Conference on Computer Vision (ECCV).

[109] Chéron G., Laptev I., Schmid C. P-cnn: Pose-based cnn features for action recognition. Proceedings of the IEEE International Conference on Computer Vision.

[110] Fernandez R.A.S., Sanchez-Lopez J.L., Sampedro C., Bavle H., Molina M., Campoy P. Natural user interfaces for human-drone multi-modal interaction. Proceedings of the 2016 International Conference on Unmanned Aircraft Systems (ICUAS).

[111] Bruce J., Perron J., Vaughan R. Ready—aim—fly! hands-free face-based HRI for 3D trajectory control of UAVs. Proceedings of the 2017 14th Conference on Computer and Robot Vision (CRV).

[112] Nagi J., Giusti A., Di Caro G.A., Gambardella L.M. Human control of UAVs using face pose estimates and hand gestures. Proceedings of the 2014 9th ACM/IEEE International Conference on Human-Robot Interaction (HRI).

[113] Nagi J., Giusti A., Gambardella L.M., Di Caro G.A. Human-swarm interaction using spatial gestures. Proceedings of the 2014 IEEE/RSJ International Conference on Intelligent Robots and Systems.

[114] Obaid M., Kistler F., Kasparavičiūtė G., Yantaç A.E., Fjeld M. How would you gesture navigate a drone? A user-centered approach to control a drone. Proceedings of the 20th International Academic Mindtrek Conference.

[115] Cauchard J.R., E J.L., Zhai K.Y., Landay J.A. Drone & me: An exploration into natural human-drone interaction. Proceedings of the 2015 ACM International Joint Conference on Pervasive and Ubiquitous Computing.

[116] Davis N., Pittaluga F., Panetta K. Facial recognition using human visual system algorithms for robotic and UAV platforms. Proceedings of the 2013 IEEE Conference on Technologies for Practical Robot Applications (TePRA).

[117] Nousi P., Tefas A. Discriminatively trained autoencoders for fast and accurate face recognition. Proceedings of the International Conference on Engineering Applications of Neural Networks.

[118] Hsu H.J., Chen K.T. Face recognition on drones: Issues and limitations. Proceedings of the First Workshop on Micro Aerial Vehicle Networks, Systems, and Applications for Civilian Use.

[119] Li Y., Scanavino M., Capello E., Dabbene F., Guglieri G., Vilardi A. (2018). A novel distributed architecture for UAV indoor navigation. Transp. Res. Procedia.

[120] Mori T., Scherer S. First results in detecting and avoiding frontal obstacles from a monocular camera for micro unmanned aerial vehicles. Proceedings of the 2013 IEEE International Conference on Robotics and Automation.

[121] Hamledari H., McCabe B., Davari S. (2017). Automated computer vision-based detection of components of under-construction indoor partitions. Autom. Constr..

[122] Loquercio A., Kaufmann E., Ranftl R., Dosovitskiy A., Koltun V., Scaramuzza D. (2019). Deep drone racing: From simulation to reality with domain randomization. IEEE Trans. Robot..

[123] Hsu H.J., Chen K.T. DroneFace: An open dataset for drone research. Proceedings of the 8th ACM on Multimedia Systems Conference.

[124] Kalra I., Singh M., Nagpal S., Singh R., Vatsa M., Sujit P. Dronesurf: Benchmark dataset for drone-based face recognition. Proceedings of the 2019 14th IEEE International Conference on Automatic Face & Gesture Recognition (FG 2019).

[125] Perera A.G., Law Y.W., Chahl J. (2019). Drone-Action: An Outdoor Recorded Drone Video Dataset for Action Recognition. Drones.

[126] Leo M., Furnari A., Medioni G.G., Trivedi M., Farinella G.M. Deep learning for assistive computer vision. Proceedings of the European Conference on Computer Vision (ECCV).

[127] Yang F., Fan H., Chu P., Blasch E., Ling H. Clustered object detection in aerial images. Proceedings of the IEEE International Conference on Computer Vision.

[128] Li C., Yang T., Zhu S., Chen C., Guan S. Density Map Guided Object Detection in Aerial Images. Proceedings of the IEEE/CVF Conference on Computer Vision and Pattern Recognition Workshops.

[129] Zhou J., Vong C.M., Liu Q., Wang Z. (2019). Scale adaptive image cropping for UAV object detection. Neurocomputing.

[130] Ledig C., Theis L., Huszár F., Caballero J., Cunningham A., Acosta A., Aitken A., Tejani A., Totz J., Wang Z. Photo-realistic single image super-resolution using a generative adversarial network. Proceedings of the IEEE Conference on Computer Vision and Pattern Recognition.

[131] Zhang P., Zhong Y., Li X. SlimYOLOv3: Narrower, faster and better for real-time UAV applications. Proceedings of the IEEE International Conference on Computer Vision Workshops.

[132] Zhao H., Zhou Y., Zhang L., Peng Y., Hu X., Peng H., Cai X. (2020). Mixed YOLOv3-LITE: A lightweight real-time object detection method. Sensors.

[133] Pedoeem J., Huang R. YOLO-LITE: A Real-Time Object Detection Algorithm Optimized for Non-GPU Computers. Proceedings of the 2018 IEEE International Conference on Big Data.

[134] Jabari S., Fathollahi F., Zhang Y. (2017). Application of Sensor Fusion to Improve Uav Image Classification. Int. Arch. Photogramm. Remote Sens. Spat. Inf. Sci..

[135] Ki M., Cha J., Lyu H. Detect and avoid system based on multi sensor fusion for UAV. Proceedings of the 2018 International Conference on Information and Communication Technology Convergence (ICTC).

[136] Półka M., Ptak S., Kuziora Ł. (2017). The use of UAV’s for search and rescue operations. Procedia Eng..

[137] Tijtgat N., Van Ranst W., Goedeme T., Volckaert B., De Turck F. Embedded real-time object detection for a UAV warning system. Proceedings of the IEEE International Conference on Computer Vision Workshops.

[138] Dollár P., Appel R., Belongie S., Perona P. (2014). Fast feature pyramids for object detection. IEEE Trans. Pattern Anal. Mach. Intell..

[139] Redmon J., Farhadi A. YOLO9000: Better, faster, stronger. Proceedings of the IEEE Conference on Computer Vision and Pattern Recognition.

[140] Friedman J., Hastie T., Tibshirani R. (2000). Additive logistic regression: A statistical view of boosting (with discussion and a rejoinder by the authors). Ann. Stat..

[141] Lygouras E., Gasteratos A., Tarchanidis K., Mitropoulos A. (2018). ROLFER: A fully autonomous aerial rescue support system. Microprocess. Microsyst..

[142] Lygouras E., Santavas N., Taitzoglou A., Tarchanidis K., Mitropoulos A., Gasteratos A. (2019). Unsupervised Human Detection with an Embedded Vision System on a Fully Autonomous UAV for Search and Rescue Operations. Sensors.

[143] Sun J., Li B., Jiang Y., Wen C.y. (2016). A camera-based target detection and positioning UAV system for search and rescue (SAR) purposes. Sensors.

[144] Ahmed A., Nagai M., Tianen C., Shibasaki R. (2008). UAV based monitoring system and object detection technique development for a disaster area. Int. Arch. Photogramm. Remote Sens. Spat. Inf. Sci..

[145] Doherty P., Rudol P. A UAV search and rescue scenario with human body detection and geolocalization. Proceedings of the Australasian Joint Conference on Artificial Intelligence.

[146] Rudol P., Doherty P. Human body detection and geolocalization for UAV search and rescue missions using color and thermal imagery. Proceedings of the 2008 IEEE Aerospace Conference.

[147] Motlagh N.H., Bagaa M., Taleb T. (2017). UAV-based IoT platform: A crowd surveillance use case. IEEE Commun. Mag..

[148] Qazi S., Siddiqui A.S., Wagan A.I. UAV based real time video surveillance over 4G LTE. Proceedings of the 2015 International Conference on Open Source Systems & Technologies (ICOSST).

[149] Gu J., Su T., Wang Q., Du X., Guizani M. (2018). Multiple moving targets surveillance based on a cooperative network for multi-UAV. IEEE Commun. Mag..

[150] Wang C., Zhang H., Yang L., Liu S., Cao X. Deep people counting in extremely dense crowds. Proceedings of the 23rd ACM international conference on Multimedia.

[151] Zhang C., Li H., Wang X., Yang X. Cross-scene crowd counting via deep convolutional neural networks. Proceedings of the IEEE Conference on Computer Vision and Pattern Recognition.

[152] Almagbile A. (2019). Estimation of crowd density from UAVs images based on corner detection procedures and clustering analysis. Geo-Spat. Inf. Sci..

[153] Bansal A., Venkatesh K. (2015). People counting in high density crowds from still images. arXiv.

[154] Shami M.B., Maqbool S., Sajid H., Ayaz Y., Cheung S.C.S. (2018). People counting in dense crowd images using sparse head detections. IEEE Trans. Circuits Syst. Video Technol..

[155] Basalamah S., Khan S.D., Ullah H. (2019). Scale driven convolutional neural network model for people counting and localization in crowd scenes. IEEE Access.

[156] Huynh V.S., Tran V.H., Huang C.C. Iuml: Inception U-Net Based Multi-Task Learning For Density Level Classification And Crowd Density Estimation. Proceedings of the 2019 IEEE International Conference on Systems, Man and Cybernetics (SMC).

[157] Chandra R., Bhattacharya U., Bera A., Manocha D. (2019). DensePeds: Pedestrian Tracking in Dense Crowds Using Front-RVO and Sparse Features. arXiv.

[158] Idrees H., Tayyab M., Athrey K., Zhang D., Al-Maadeed S., Rajpoot N., Shah M. Composition loss for counting, density map estimation and localization in dense crowds. Proceedings of the European Conference on Computer Vision (ECCV).

[159] Singh A., Patil D., Omkar S. Eye in the sky: Real-time Drone Surveillance System (DSS) for violent individuals identification using ScatterNet Hybrid Deep Learning network. Proceedings of the IEEE Conference on Computer Vision and Pattern Recognition Workshops.

[160] Perera A.G., Law Y.W., Ogunwa T.T., Chahl J. (2020). A Multiviewpoint Outdoor Dataset for Human Action Recognition. IEEE Trans. Hum. Mach. Syst..

[161] Tripathi G., Singh K., Vishwakarma D.K. (2019). Convolutional neural networks for crowd behaviour analysis: A survey. Vis. Comput..

[162] Bour P., Cribelier E., Argyriou V. (2019). Crowd behavior analysis from fixed and moving cameras. Multimodal Behavior Analysis in the Wild.

[163] Mueller M., Smith N., Ghanem B. A benchmark and simulator for uav tracking. Proceedings of the European Conference on Computer Vision.

[164] Zhu P., Wen L., Du D., Bian X., Ling H., Hu Q., Nie Q., Cheng H., Liu C., Liu X. Visdrone-det2018: The vision meets drone object detection in image challenge results. Proceedings of the European Conference on Computer Vision (ECCV).

[165] Du D., Zhu P., Wen L., Bian X., Lin H., Hu Q., Peng T., Zheng J., Wang X., Zhang Y. VisDrone-DET2019: The Vision Meets Drone Object Detection in Image Challenge Results. Proceedings of the IEEE International Conference on Computer Vision (ICCV) Workshops.

[166] Zhu P., Du D., Wen L., Bian X., Ling H., Hu Q., Peng T., Zheng J., Wang X., Zhang Y. VisDrone-VID2019: The Vision Meets Drone Object Detection in Video Challenge Results. Proceedings of the 2019 IEEE/CVF International Conference on Computer Vision Workshop (ICCVW).

[167] Du D., Qi Y., Yu H., Yang Y., Duan K., Li G., Zhang W., Huang Q., Tian Q. The unmanned aerial vehicle benchmark: Object detection and tracking. Proceedings of the European Conference on Computer Vision (ECCV).

[168] Robicquet A., Sadeghian A., Alahi A., Savarese S. Learning social etiquette: Human trajectory understanding in crowded scenes. Proceedings of the European Conference on Computer Vision.

[169] Bozcan I., Kayacan E. AU-AIR: A Multi-Modal Unmanned Aerial Vehicle Dataset for Low Altitude Traffic Surveillance. http://xxx.lanl.gov/abs/2001.11737.

[170] Chan A.B., Liang Z.S.J., Vasconcelos N. Privacy preserving crowd monitoring: Counting people without people models or tracking. Proceedings of the 2008 IEEE Conference on Computer Vision and Pattern Recognition.

[171] Hirzer M., Beleznai C., Roth P.M., Bischof H. Person re-identification by descriptive and discriminative classification. Proceedings of the Scandinavian Conference on Image Analysis.

[172] Layne R., Hospedales T.M., Gong S. Investigating open-world person re-identification using a drone. Proceedings of the European Conference on Computer Vision.

[173] Kumar S., Yaghoubi E., Das A., Harish B., Proença H. (2020). The P-DESTRE: A Fully Annotated Dataset for Pedestrian Detection, Tracking, Re-Identification and Search from Aerial Devices. arXiv.

[174] Xu Y., Fu M., Wang Q., Wang Y., Chen K., Xia G.S., Bai X. (2020). Gliding vertex on the horizontal bounding box for multi-oriented object detection. IEEE Trans. Patt. Anal. Mach. Intel..

[175] Li K., Wan G., Cheng G., Meng L., Han J. (2020). Object detection in optical remote sensing images: A survey and a new benchmark. ISPRS J. Photogramm. Remote Sens..

[176] Zheng K., Wei M., Sun G., Anas B., Li Y. (2019). Using vehicle synthesis generative adversarial networks to improve vehicle detection in remote sensing images. ISPRS Int. J. Geo-Inf..

[177] Lin H., Zhou J., Gan Y., Vong C.M., Liu Q. (2020). Novel Up-scale Feature Aggregation for Object Detection in Aerial Images. Neurocomputing.

[178] Tang T., Zhou S., Deng Z., Zou H., Lei L. (2017). Vehicle detection in aerial images based on region convolutional neural networks and hard negative example mining. Sensors.

[179] Cheng H.Y., Weng C.C., Chen Y.Y. (2011). Vehicle detection in aerial surveillance using dynamic Bayesian networks. IEEE Trans. Image Process..

[180] Gleason J., Nefian A.V., Bouyssounousse X., Fong T., Bebis G. Vehicle detection from aerial imagery. Proceedings of the 2011 IEEE International Conference on Robotics and Automation.

[181] Cheng M.M., Zhang Z., Lin W.Y., Torr P. BING: Binarized normed gradients for objectness estimation at 300fps. Proceedings of the IEEE Conference on Computer Vision and Pattern Recognition.

[182] Chen X., Xiang S., Liu C.L., Pan C.H. (2014). Vehicle detection in satellite images by hybrid deep convolutional neural networks. IEEE Geosci. Remote Sens. Lett..

[183] Sommer L.W., Schuchert T., Beyerer J. Fast deep vehicle detection in aerial images. Proceedings of the 2017 IEEE Winter Conference on Applications of Computer Vision (WACV).

[184] Deng Z., Sun H., Zhou S., Zhao J., Zou H. (2017). Toward fast and accurate vehicle detection in aerial images using coupled region-based convolutional neural networks. IEEE J. Sel. Top. Appl. Earth Obs. Remote Sens..

[185] Liu K., Mattyus G. (2015). Fast multiclass vehicle detection on aerial images. IEEE Geosci. Remote Sens. Lett..

[186] Zhu H., Chen X., Dai W., Fu K., Ye Q., Jiao J. Orientation robust object detection in aerial images using deep convolutional neural network. Proceedings of the 2015 IEEE International Conference on Image Processing (ICIP).

[187] Abdollahi A., Pradhan B., Shukla N., Chakraborty S., Alamri A. (2020). Deep Learning Approaches Applied to Remote Sensing Datasets for Road Extraction: A State-Of-The-Art Review. Remote Sens..

[188] Mnih V., Hinton G.E. Learning to detect roads in high-resolution aerial images. Proceedings of the European Conference on Computer Vision.

[189] Zhang Z., Liu Q., Wang Y. (2018). Road extraction by deep residual u-net. IEEE Geosci. Remote Sens. Lett..

[190] Ronneberger O., Fischer P., Brox T. U-net: Convolutional networks for biomedical image segmentation. Proceedings of the International Conference on Medical Image Computing and Computer-Assisted Intervention.

[191] Gao L., Song W., Dai J., Chen Y. (2019). Road extraction from high-resolution remote sensing imagery using refined deep residual convolutional neural network. Remote Sens..

[192] Benediktsson J.A., Pesaresi M., Amason K. (2003). Classification and feature extraction for remote sensing images from urban areas based on morphological transformations. IEEE Trans. Geosci. Remote Sens..

[193] Zhong P., Wang R. (2007). A multiple conditional random fields ensemble model for urban area detection in remote sensing optical images. IEEE Trans. Geosci. Remote Sens..

[194] Kovács A., Szirányi T. (2012). Improved harris feature point set for orientation-sensitive urban-area detection in aerial images. IEEE Geosci. Remote Sens. Lett..

[195] Harris C.G., Stephens M. (1988). A combined corner and edge detector. Alvey Vision Conference.

[196] Manno-Kovacs A., Sziranyi T. (2015). Orientation-selective building detection in aerial images. ISPRS J. Photogramm. Remote Sens..

[197] Saito S., Yamashita T., Aoki Y. (2016). Multiple object extraction from aerial imagery with convolutional neural networks. Electron. Imaging.

[198] Hayrapetyan N., Hakobyan R., Poghosyan A., Gabrielyan V. (2016). Border Surveillance Using UAVs with Thermal Camera. Meeting Security Challenges Through Data Analytics and Decision Support.

[199] Byerlay R.A., Nambiar M.K., Nazem A., Nahian M.R., Biglarbegian M., Aliabadi A.A. (2020). Measurement of land surface temperature from oblique angle airborne thermal camera observations. Int. J. Remote Sens..

[200] Xia G.S., Bai X., Ding J., Zhu Z., Belongie S., Luo J., Datcu M., Pelillo M., Zhang L. DOTA: A large-scale dataset for object detection in aerial images. Proceedings of the IEEE Conference on Computer Vision and Pattern Recognition.

[201] Razakarivony S., Jurie F. (2016). Vehicle detection in aerial imagery: A small target detection benchmark. J. Vis. Commun. Image Represent..

[202] Mundhenk T.N., Konjevod G., Sakla W.A., Boakye K. A large contextual dataset for classification, detection and counting of cars with deep learning. Proceedings of the European Conference on Computer Vision.

[203] Andriluka M., Roth S., Schiele B. People-tracking-by-detection and people-detection-by-tracking. Proceedings of the 2008 IEEE Conference on Computer Vision and Pattern Recognition.

[204] Liew C.F., Yairi T. (2020). Companion Unmanned Aerial Vehicles: A Survey. arXiv.

